# Solution-Processed Metal-Oxide Nanoparticles to Prevent The Sputtering Damage in Perovskite/Silicon Tandem Solar Cells

**DOI:** 10.1021/acsami.5c00090

**Published:** 2025-03-10

**Authors:** Erica Magliano, Francesco Di Giacomo, Harshavardhan Reddy Sathy, Shirin M. Pourmotlagh, Gemma Giliberti, David Becerril Rodriguez, Giuseppe Ammirati, Paolo Mariani, Francesca Zarotti, Fabio Matteocci, Marco Luce, Iurie Usatii, Eugenia Bobeico, Marco Della Noce, Antonio Cricenti, Federica Cappelluti, Lucia V. Mercaldo, Paola Delli Veneri, Aldo Di Carlo

**Affiliations:** a CHOSE (Centre for Hybrid and Organic Solar Energy), Department of Electronic Engineering, Tor Vergata University of Rome, Via del Politecnico 1, Rome 00118, Italy; b Department of Electronics and Telecommunication, Politecnico di Torino, Corso Duca degli Abruzzi 24, Turin 10129, Italy; c Istituto di Struttura della Materia (CNR-ISM) National Research Council, via del Fosso del Cavaliere 100, Rome 00133, Italy; d ENEA − Portici Research Center, P.le Enrico Fermi 1, Portici (Naples) 80055, Italy

**Keywords:** protective buffer layers, sputtering damage, semitransparent, perovskite/silicon tandem solar cells, AZO nanoparticles, light management

## Abstract

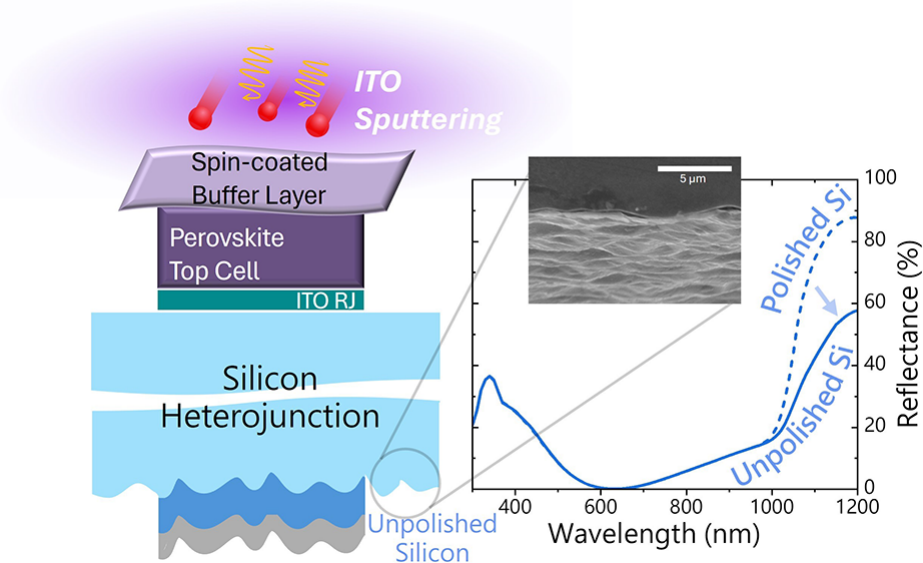

Semitransparent perovskite solar cells (ST-PSCs) for tandem applications typically use a buffer layer deposited via atomic layer deposition (ALD) to protect the cell stack from the damage induced by the sputtering of the transparent electrode. Here, we present a simple yet effective solution-processed buffer layer based on metal-oxide nanoparticles to mitigate sputter-induced damage. We exploit this strategy in a monolithic tandem integrating the optimized ST-PSC on a polished front-side/unpolished rear-side *p*-type silicon heterojunction (SHJ) solar cell. The intrinsic roughness on the backside significantly boosts the absorption, thus suppressing the need for a dedicated texturization step and leading to a final maximum efficiency of 25.3%. Our findings highlight the potential of solution-processed buffer layers as a practical and scalable solution to mitigate the sputtering damage, as well as the potential of silicon wafers with an unpolished rear surface for enhanced photocurrent.

## Introduction

Solar energy has emerged as a pivotal solution in the pursuit of sustainable and renewable energy sources. Among various solar cell technologies, perovskite/silicon tandem solar cells have garnered considerable attention due to their potential to overcome the Shockley–Queisser limit of single-junction devices.^1^ Metal-halide perovskite materials offer excellent optoelectronic properties and a tunable band gap. By combining a perovskite top cell, tuned to absorb the high energy photons, with a silicon bottom cell absorbing the complementary low energy region of the solar spectrum, improved efficiency can be achieved, thanks to reduced thermalization losses. Recent advancements have yielded impressive results, with a world record power conversion efficiency (*PCE*) of 34.6%.^2,3^

The optimization of the semitransparent perovskite top cell in terms of electrical properties and optical transparency is crucial for achieving high efficiency in the tandem device.^4^ The top component in perovskite/Si tandem solar cells is a semitransparent perovskite solar cell (ST-PSC), where a transparent electrode is needed to ensure proper light absorption through the device. Transparent conductive oxides (TCOs), such as indium-doped tin oxide (ITO), aluminum-doped zinc oxide (AZO), indium-doped zinc oxide (IZO), represent the most common transparent electrodes employed in ST-PSCs, due to their high transparency and high electrical mobility.^5^ TCO deposition usually occurs through a sputtering method, which is framed in the physical vapor deposition (PVD) techniques. Sputtering represents the most preferable deposition method for TCOs owing to its ability to produce superior film quality, as well as its compatibility with large-scale production.^6,7^ During sputtering, energetic ions bombard the target material, causing atoms to be ejected and deposited onto a substrate to form a thin film. The process involves the generation of plasma, which is a highly energetic state of matter consisting of charged particles including ions and electrons. The energetic particles could hit the substrate, resulting in the dissociation of chemical bonds if the energy of the particles is higher than the bond-dissociation energy. Additionally, UV radiation emitted from the plasma can degrade organic materials, such as perovskite or an exposed electron- or hole-transport layer (ETL or HTL), through processes like photooxidation or photochemical degradation. Moreover, the energy transferred to the substrate during the deposition process can increase the substrate temperature. Excessive heat may induce thermal stress and cause damage to the perovskite layer or other organic layers within the solar cell structure. This can lead to the decomposition of organic materials, alteration of crystal structures, and degradation of interfaces, ultimately affecting the device performance and stability.^5,8−10^

To mitigate damage caused by sputtering, it is crucial to carefully control the sputtering parameters, such as pressure, deposition rate, deposition time, substrate temperature, and target-to-substrate distance, where possible.^11,12^ However, due to sputtering system geometry or limitations, a so-called “soft” sputtering deposition process cannot always be implemented, or the deposition rate might be impractically slow or the reproducibility might be compromised. A possible solution is the application of protective buffer layers (PBLs) to shield the perovskite and the transporting layers from direct exposure to ion bombardment and UV plasma radiation. The inclusion of a PBL favors device robustness, enhancing thermal and environmental stability.^13−15^

The most common PBL in *p-i-n* (also known as “inverted”) configuration is a layer of SnO_2_ deposited by atomic layer deposition (ALD).^16,17^ A bilayer of ZnO/Al_2_O_3_ prepared using ALD has also shown promise as a buffer layer for sputtering protection in ST-PSCs.^18^ However, the extremely slow deposition rate of ALD represents a significant roadblock to its potential industrial use.^19^ This is compounded by the expensive precursors and the poor efficiency in material utilization, which further hinder the process of industrialization.^20,21^ Moreover, the ALD-SnO_2_ process requires fine customization in order to prevent degradation of perovskite film and exposed layers.^21−23^ In this context, solution-processed PBLs represent a valid cost-effective and simple alternative. In this work, we focused on the development of a simple solution process (spin-coating) method for PBL deposition in *p-i-n* ST-PSCs. We tested a series of commercial nanoparticle dispersions based on metal oxides (i.e., ZnO, Al-doped ZnO (AZO), and SnO_2_), providing a complete overview of the effects of the different solutions in the *p-i-n* configuration. We investigated the chemical compatibility and energetic alignment of the various dispersions by evaluating the device performance of static and dynamic deposited PBL-based opaque devices. This evaluation was fundamental for proper PBL deposition and for subsequent translation into semitransparent devices. The AZO-based PBLs exhibited superior performance, particularly in terms of resilience to sputtering damage, enhanced crystallinity, and improved UV-shielding properties. The optimization of the PBL deposition led to the fabrication of fully solution-processed (electrodes excluded) ST-PSCs, achieving the maximum *PCE* of 18.1%, with open-circuit voltage (*V*_OC_) and fill factor (*FF*) comparable with the opaque counterpart. Furthermore, we demonstrated that this approach can be translated into monolithic perovskite/silicon tandem solar cells. To the best of the authors’ knowledge, the application of solution-processed AZO as a buffer layer in a perovskite/silicon tandem is unprecedented. Additionally, our work is among the first to use solution-processed buffer layers in tandem, avoiding the use of ALD-SnO_2_.^12,24^

For the bottom component, we used silicon heterojunction (SHJ) solar cells fabricated from *p*-type silicon wafers with unconventional rear-side roughness as a low-cost simplified alternative to the conventional micrometer-range random pyramidal texture obtained via wet etching. Fabricating the perovskite top cell on both-side pyramidally textured silicon cells in principle leads to superior light management and impressive device performance, achieving record *PCE*s.^17,25^ The efficient light in-coupling and light trapping determine a high photocurrent over 20 mA cm^–2^ in both the subcells in tandem configuration.^26,27^ Nevertheless, the layer conformality must be fulfilled in order to avoid shunt paths. Moreover, the perovskite growth and optical properties strongly depend on the morphology of silicon texturing. The responses of photoluminescence (*PL*) spectral intensity and quasi-Fermi level splitting (QFLS) are predominantly shaped by the underlying texture, especially with large (5 μm pyramids) texture schemes. A different halide distribution was disclosed in the perovskite film, with the presence of a Br-rich region in the valleys leading to band gap heterogeneities over the absorber layer.^28,29^ Impressive results can be achieved also with back-side textured and front-side flat silicon, with the latter undergoing a single-side polishing process.^16^ In this case, the addition of light management films on the top electrode aids in reducing reflection.^30,31^ However, single-side texturing requires the protection of the silicon front side. Surface decoupling is not a straightforward approach, as it can be time-consuming and can hinder high-throughput manufacturing due to the increased number of steps. The application and the subsequent removal of a protective cover on the front side may increase surface recombination and induce *V*_OC_ losses in the final device.^32^ More in general, the texturing processes involve a significant waste of precious device-grade silicon and chemicals from the employed solution.^33^ Preserving the intrinsic roughness of the silicon wafer, after the ingot slicing, on the rear side can represent a valid cost-effective alternative, which can further lower the levelized cost of electricity (LCOE).^34^ Even though the standard pyramidal texturing offers optimal light management, we demonstrated that the roughness of the unpolished silicon wafer on the rear side provides significant light trapping.

Additionally, it is noteworthy that the commonly employed silicon wafers for SHJs are *n*-type, due to their higher bulk carrier lifetime with respect to *p*-type, leading to higher achieved efficiencies. However, *p*-type Si-based SHJs could potentially lead to similar performances as compared to *n*-type Si-based SHJ,^35−37^ with the advantage of an estimated 8–10% lower wafer cost.^34,38,39^

Here, we employed SHJ manufactured from polished front-side/unpolished rear-side *p*-type Si-wafers for monolithic perovskite/silicon tandem fabrication. The resulting two-terminal tandem showed a *PCE* of 23.2% with solution-processed perovskite and 25.3% with the two-step hybrid perovskite.

## Results and Discussion

In this study, we investigated three types of metal oxides, namely, ZnO, AZO (Al:ZnO), and SnO_2_ nanoparticle solutions, and obtained various nanoparticle formulations for each oxide. Table S1 provides a summary of the materials used in the study, including their respective code names, particle sizes, work functions, concentrations (weight percentage, wt %), solvents, viscosity, and coating applicability. The ZnO dispersion consists of four different types of formulations (N-10, N-10-Flex, N-11, and N-12), whereas Al:ZnO (AZO) solutions comprise three formulations (N-20X-Flex, N-21X, and N-21X-Flex). The code of the employed SnO_2_ suspension was N-30. The work functions range from 3.9 to 4.3 eV, which can ensure a proper energetic alignment with RF-sputtered ITO, showing a work function (ϕ) of 5.0 eV (measured with Kelvin probe system), as well as with Cu (ϕ ≈ 4.7 eV).^40−42^ The concentration is consistently 2.5 wt % for most of the nanoparticle dispersions. The solution (ZnO N-12) with 5 wt % was diluted to 2.5 wt % to perform a fair comparison. The coating applicability encompasses spin coating, blade coating, and slot-die-coating techniques, favoring potential upscaling. Here, the spin-coating technique was employed for PBL deposition.

**Figure 1:**
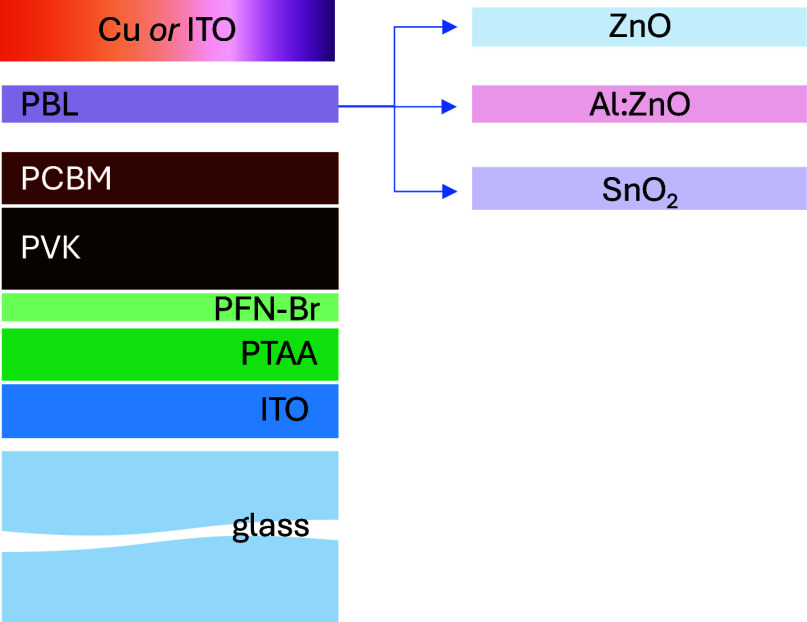
Device architecture of the opaque PSCs with the different PBL employed in this work. In the semitransparent devices, Cu was replaced with an ITO top electrode.

To assess the compatibility and investigate the effects of functional metal oxide-based PBL in an inverted PSC structure, the layers were first tested in the opaque architecture and then translated to semitransparent devices. Perovskite solar cells with an inverted device configuration of glass/ITO/PTAA/PFN-Br/Cs_0.05_MA_0.14_FA_0.81_PbI_2.7_Br_0.3_/PC_61_BM/PBL/Cu (Figure 1) were employed as control devices, where solution-processed PTAA and PC_61_BM layers act as the hole- and electron-transport layers, respectively. The perovskite band gap is 1.6 eV.^43^ To investigate potential solvent interactions with the underlying layers, both static and dynamic coating depositions were employed for each solution. In the static deposition method, the dispersion was dropped onto the device and left spreading for 5 s before spinning. Conversely, in the dynamic deposition method, the solution was dropped onto the rotating device, reducing the interaction time with the underlying layers. The static or dynamic spin-coating technique determines the solvent evaporation speed. Additionally, the deposition method affects the interaction with the underlying layers, which could lead to the etching effect and underlayer dissolution in the worst cases, resulting in the formation of pores or pinholes.^44^

**Figure 2:**
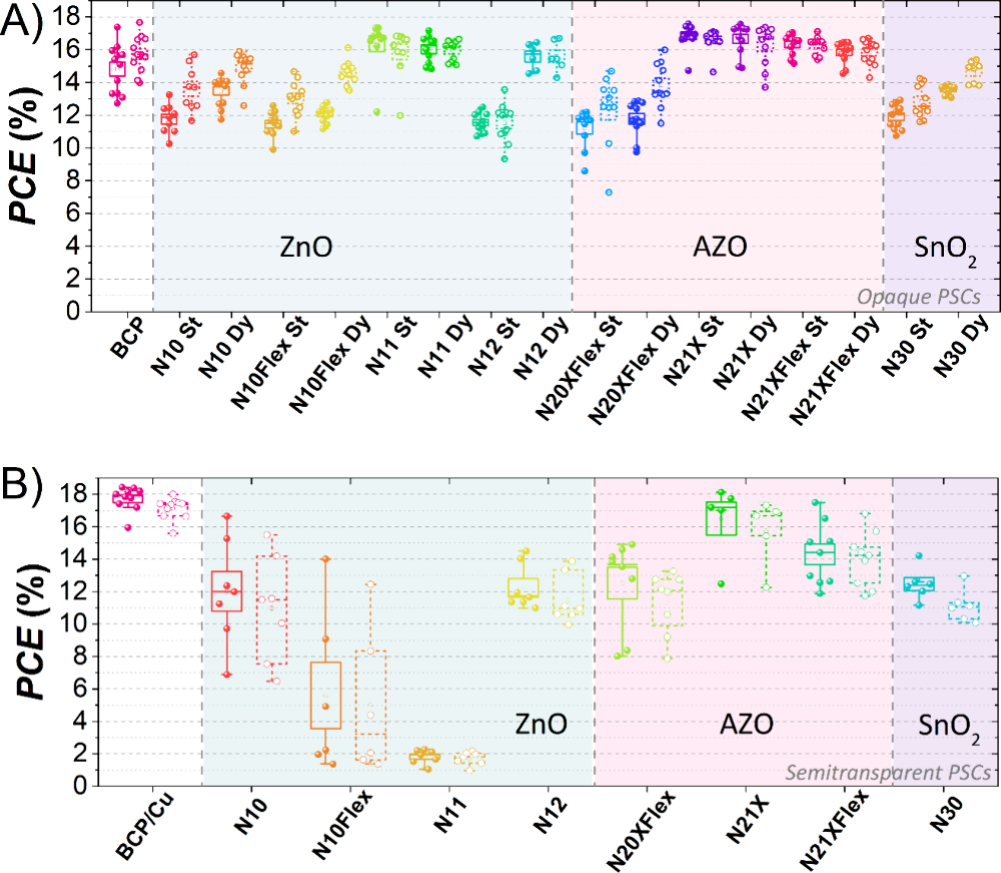
(A) Statistical distribution of the *PCE* in reverse (closed dot) and forward (open dots) scan of perovskite solar cells (glass/ITO/PTAA/PFN-Br/Cs_0.05_MA_0.14_FA_0.81_PbI_2.7_Br_0.3_/PC_61_BM/PBL/Cu) with various solution-processed PBLs deposited via static (St) and dynamic (Dy) coating. (B) *PCE* of semitransparent perovskite solar cells (glass/ITO/PTAA/PFN-Br/Cs_0.05_MA_0.14_FA_0.81_PbI_2.7_Br_0.3_/PC_61_BM/PBL/ITO) with different solution-processed PBLs and RF-sputtered ITO.

The statistical distribution of *PCE* of the PBL-based devices in comparison with the reference devices with BCP is reported in Figure 2A. *V*_OC_, FF, and short-circuit current density (*J*_SC_) are reported in the SI (Figure S2, Table S2).

The investigation of ZnO-based nanoparticle dispersions revealed an interesting trend in their performance with respect to the coating method. ZnO N-11 solution demonstrated comparable *PCE*s when coated using both static and dynamic methods as well as a reduced hysteresis as compared to the other ZnO dispersions. Among all the ZnO-tested solutions, N-11 showcased marginally better or comparable performance to the reference devices. The improved *J*_SC_ and FF values of these devices contributed to the observed performance enhancement. Except for N-11, all other ZnO-based solutions exhibited superior performance when subjected to dynamic coating compared to static coating. An *FF* decrease was observed in the static method-based devices. As an example, ZnO N-10 film morphology was analyzed (Figure S3A), disclosing the presence of some voids in the statically spin-coated film, whereas a more uniform and homogeneous film was obtained in the dynamic spin-coating case. The static dispense of the solution can cause a partial etching of the underlying layers, causing the formation of bubbles in the PBL solution and subsequently generating gaps within the layer. This can explain the *FF* losses in the static method.

**Figure 3:**
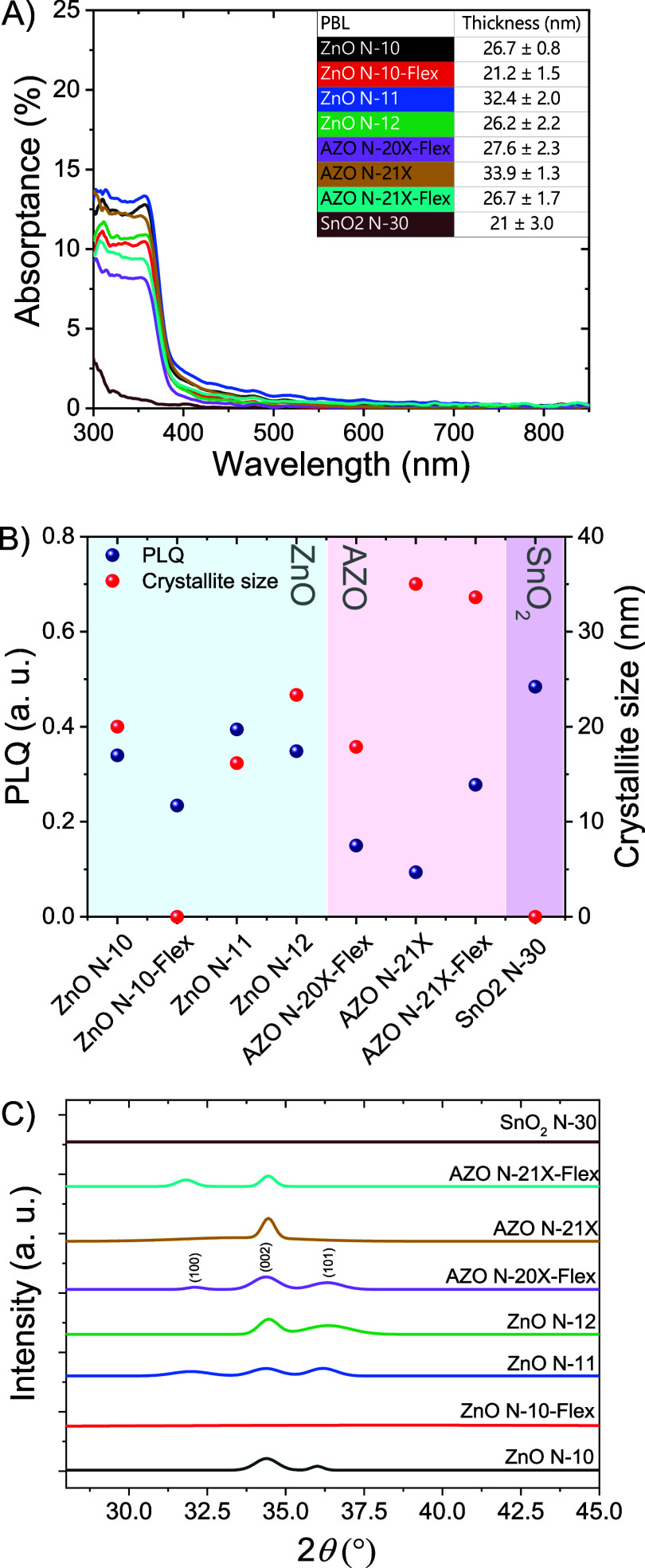
(A) Absorptance spectra of ZnO (N-10, N-10-Flex, N-11, N-12), AZO (N-20X-Flex, N-21X, N-21X-Flex) and SnO_2_ (N30) as bare films. The thickness of each layer is also reported in the table. (B) On the left axis (blue dots), PLQ determined from the PL measurement on the PSC stack fabricated to the PBL (ZnO, AZO or SnO_2_), before and after UV-plasma treatment. On the right axis, crystallite size was obtained with the Scherrer equation from the XRD patterns in (C) on the bare PBLs. (C) XRD patterns of the bare PBLs deposited on glass soda lime.

In the case of AZO-based solutions, the dynamic PBL deposition proved to be beneficial in N-20X-Flex-based devices, showing a higher performance. This finding suggests that N-20X-Flex exhibits a strong interaction with the underlying layers. However, a significant hysteresis was observed (see Figure 2A), which could be attributed to poor charge extraction properties or increased trap-assisted charge recombination.^45^ On the other hand, the remaining AZO-based solutions did not exhibit a similar trend, indicating limited interaction with the beneath layers. While the N-20X-Flex-based devices showcased lower performance than the reference device, both N-21X and N-21X-Flex-based PSCs exhibited significant improvements in terms of *PCE*, *J*_SC_, as well as *FF*, when compared to the reference. The enhanced performance of these dispersions can be attributed to the improved *J*_SC_ and *FF*, which result from the increased crystallite size observed in Figure 3B and discussed later in the study. The devices based on AZO N-21X showed similar performances between static and dynamic spin-coating methods with a higher *FF* in the devices based on the dynamic processing of the nanoparticles. This can be attributed to the improved uniformity observed in the case of dynamically deposited AZO, as shown in Figure S3B. In the case of static spin-coating, some bubble-like voids are visible on the film, which can be due to the etching of the underlying layers and/or improper solvent evaporation. These voids are shallow and, therefore, do not form direct pathways, resulting in no detrimental impact on the performance of the device. On the other hand, N-21X-Flex-based PSCs exhibited slightly higher *PCE* in the static method case, mainly due to *J*_SC_. The film morphology (Figure S3C) disclosed the presence of some surface voids in both cases, which denote similar solvent evaporation rates of AZO N-21X-Flex during static and dynamic deposition methods.

The SnO_2_-based devices exhibited a significant decrease in FF, showing inferior performance compared to the other metal oxide-based devices.

This analysis on opaque devices revealed the effectiveness of dynamic spin-coating deposition, which drastically affected the performance in some cases (i.e., ZnO N-10, ZnO N-10-Flex, ZnO N-12, AZO N-20X-Flex, and SnO*_2_* N-30). Therefore, the dynamic spin-coating technique was selected for PBL deposition.

As previously mentioned, the primary sources of damage during the sputtering process are the bombardment of energetic particles and UV radiation emitted from the plasma. The UV blocking capability of the nanoparticle-based layers was evaluated through the film absorptance measurements (Figure 3A). Unlike ZnO and AZO, the SnO_2_ N-30 layer is transparent to UV light, allowing a direct exposure of the underlying layers to UV radiation during the sputtering process. In contrast, ZnO and AZO layers show an absorptance between 9% and 20% in the UV wavelength range (300–380 nm), making them effective for UV protection. The impact of UV exposure during the sputtering process on the PBLs was further evaluated by masking the films with a glass microslide and subsequently exposing the substrates to the UV plasma. The absorptance spectra (Figure S4) demonstrated that UV radiation does not induce any optical changes in the oxides, thereby preserving consistent optical properties throughout the process.

To investigate the direct sputtering-induced damage from UV exposure during ST-PSC fabrication, we realized samples processed up to the PBL (sample structure: glass/ITO/PTAA/PFN-Br/perovskite/PC_61_BM/PBL). We evaluated the shielding property of the PBLs through photoluminescence (*PL*) spectra by determining the PL quenching (PLQ) as described in the SI. In all cases, a quenching of the PL spectrum was observed upon UV-plasma exposure, as shown in Figure 3B, indicating an increment of defects acting as nonradiative recombination centers. However, AZO-based samples and ZnO N-10-Flex demonstrated greater resilience to sputtering damage compared to the other oxides, with reduced quenching and PLQ < 0.3. Notably, AZO N-21X displayed the lowest PLQ of 0.1, highlighting its superior mitigation of sputtering-induced damage.

Additionally, XRD patterns of the ZnO, AZO, and SnO_2_ films were analyzed, revealing three major peaks at 31.8, 34.4, and 36.2°, corresponding to the (100), (002), and (101) planes, respectively (Figure 3C).^46,47^ While SnO_2_ and ZnO N-10-Flex exhibited an amorphous structure, other samples displayed polycrystalline behavior. Among these, AZO N-21X uniquely demonstrated preferential vertical crystal growth along the (002) plane (*c*-axis). Preferential vertical crystal growth, without significant lateral orientation, enhances charge transfer along a single direction, which is advantageous for improving charge transport efficiency. This occurs because the strong alignment of the crystallites along the *c*-axis minimizes the scattering of charge carriers at crystallite boundaries and crystal defects.^48^

The crystallite size was calculated using the Scherrer equation, which associates the grain size of a crystal to the full width half maximum (FWHM) of a peak in the related diffraction pattern as follows:
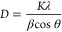
1where *D* is the crystallite size, λ is the X-ray wavelength and it is equal to 1.541 Å, β is the FWHM in radians, θ is the Bragg angle, *K* is known as the shape factor which is determined by the crystal shape and the peak width.^49^ To evaluate the height of crystal planes perpendicular to the substrate, *K* equals 0.9 using the FWHM as peak width.^50^ Here, the crystallite size was determined from the FWHM of the high-intensity peak, which is associated with the (002) plane. Larger crystallites indicate a higher structural coherence within the polycrystalline material, resulting in a lower density of grain boundaries and leading to a more efficient pathway for charge carriers.^48,51^ The data (Figure 3B) confirm the superior crystallinity of AZO N-21X as compared with the other layers.

In summary, the analysis identified AZO N-21X as the most promising buffer layer, owing to its superior performance in mitigating sputtering-induced damage, effective UV shielding, and optimized crystal structure for enhanced charge transport.

To evaluate the impact of the PBLs on device performance, ZnO, AZO, and SnO_2_ layers were tested on ST-PSCs.

The analysis performed on opaque devices was extended to ST-PSCs, where Cu was replaced with RF-sputtered ITO. We considered the glass/ITO/PTAA/PFN-Br/Cs_0.05_MA_0.14_FA_0.81_PbI_2.7_Br_0.3_/PC_61_BM/Metal-Oxide-NPs/ITO/Cu-fork architecture by varying the metal-oxide NPs and adding a Cu charge collecting structure (Cu-fork) on the side of the sputtered ITO layer. To assess the possible reduction of performance with respect to opaque devices, we assumed the glass/ITO/PTAA/PFN-Br/Cs_0.05_MA_0.14_FA_0.81_PbI_2.7_Br_0.3_/PC_61_BM/BCP/Cu stack as the control device. The electrical performance for different buffer layers is reported in Figure 2B (*J*_SC_, *V*_OC_, and FF can be found in Figure S6 and numerical values in Table S3). Here, we do not report the ST-PSCs based on ITO/BCP (without PBL) since the *J–V* showed the typical *S*-shape, indicating significant sputtering damage (see Figure S7A).^12^

As regards the ZnO PBLs, ST-PSCs based on N-10 showed the best performance, with an *FF* up to 75.7%, which could be ascribed to the best trade-off among PLQ, crystallinity, and UV protection. Nonetheless, the statistical distributions of the electrical parameters exhibited a large dispersion, indicating poor reproducibility. Similarly, a huge spread can be observed in the statistics of N-10-Flex-based ST-PSCs, yet with a lower efficiency. ZnO N-11-based ST-PSCs showed extremely reduced electrical parameters, manifesting the typical *S*-shape and suggesting strong sputtering damage (Figure S7A), as expected from the high PLQ. The best reproducibility was observed in the ZnO N-12-based ST-PSCs, even though poor *FF* and *J*_SC_ were measured. Regarding SnO_2_, the devices presented poor *J*_SC_ and *FF*, as already shown in the opaque case, and due to the amorphous structure, as well as a high PLQ effect. In the case of AZO layers, ST-PSCs followed a trend similar to that of the opaque case, showing a superior *PCE* of 18.1% in AZO N-21X-based devices, close to the best opaque reference device with a *PCE* of 18.4%. A slightly lower performance was observed in the N-21X-Flex case, whereas a significant *FF* decay was shown in the AZO N-20X-Flex-based ST-PSCs. Devices incorporating AZO-based PBLs exhibited higher *J*_SC_ values compared to those with other oxides, aligning with the PLQ results and indicating a reduction in nonradiative recombination losses. The improved charge transport and lower defect density in AZO-based samples reinforce the PL findings. Thus, the superior performance of the AZO-based ST-PSCs can be attributed to a stronger resilience to sputtering damage as well as to the superior crystallinity along one preferential direction, as previously demonstrated.

Considering the superior performance and improved resilience to sputtering damage, the AZO N-21X layer was selected as the optimal PBL for our semitransparent stack, resulting in a fully solution-processed ST-PSC.

**Figure 4:**
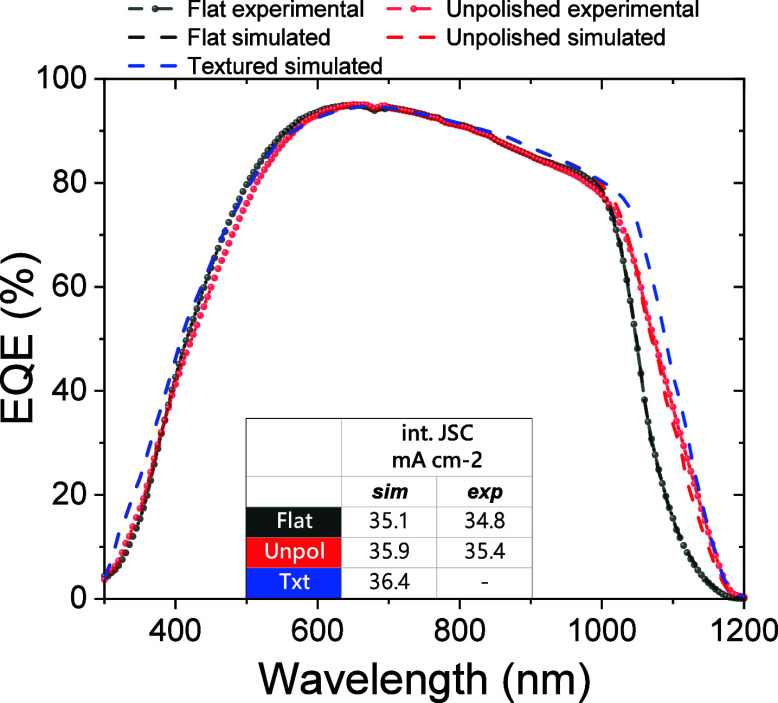
External quantum efficiency (*EQE*) of reference SHJ cells prepared from double-sided polished (black dash-dot line, denoted as “flat”) and single-sided polished (rough surface at the rear side, red dash-dot line, denoted as “unpolished”) Si wafers. The simulated *EQE* are also depicted with black and red dashed line curves, respectively. The simulated *EQE* of a textured backside SHJ with a regular pyramidal texture with a pyramid height of 5 μm (σ_RMS_ = 1241 nm) is reported for comparison (dashed blue line). The relative integrated current densities, extracted from the simulated (“sim”) or the experimental (“exp”) curves are reported in the table.

To fabricate monolithic tandem solar cells, single-side polished (polished front-side/unpolished rear-side) *p*-type Si wafer-based SHJ cells were employed as bottom cells. The SHJ component, as standalone device, had an efficiency of ∼17.7%, measured on 2 × 2 cm^2^ devices completed with AZO front electrode and metal collection grid (indium-free design, as shown in Figure S8A).^52^ The rear-side roughness (σ_RMS_ = 241 nm, Figure S8B–E) enhances the absorption in the near-infrared region, boosting the photocurrent of the SHJ cell of approximately 0.6 mA cm^–2^ as compared with a double-side polished SHJ (Figure 4). Aiming at further exploring the effect of the back-side roughness compared to a standard pyramidal texture, we performed optoelectronic simulations with the Setfos Software.^53^ As shown in Figure 4, the simulated *EQE*s for SHJ devices with flat and unpolished rear surfaces well matched the corresponding experimental spectra, while the pyramidal texture with a pyramid height of 5 μm (σ_RMS_ = 1180 nm, Figure S8F) would only provide a minor additional improvement (blue curve).

**Figure 5:**
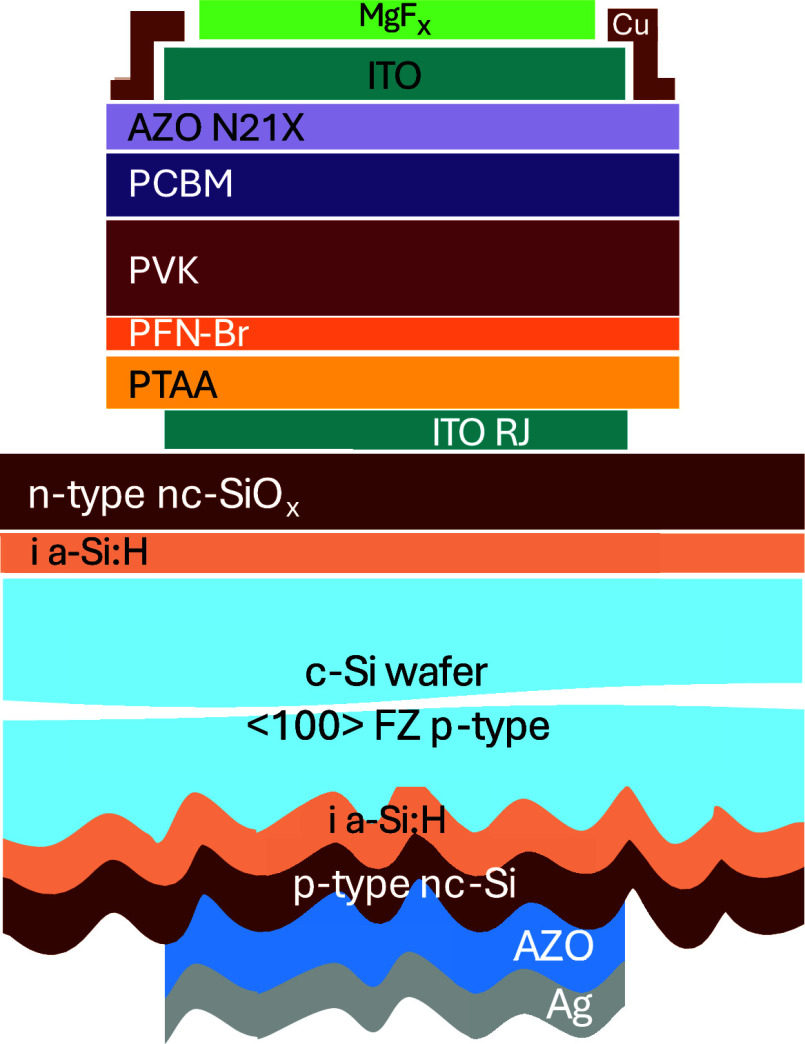
Schematics of the monolithic perovskite/silicon tandem solar cell stack.

The optimized semitransparent stack (PTAA/PFN-Br/PVK/PEACl/PCBM/AZO-N-21X/ITO/Cu frame) was translated into monolithic tandem. Moreover, a layer of MgF_2_ acting as antireflective coating was thermally evaporated on top (Figure 5). The resulting monolithic perovskite/silicon tandem exhibited a device efficiency of 23.2%, showing an *FF* of 74.6% and a *V*_OC_ of 1.75 V.

In view of a translation on fully textured or fully rough bottom cells, a two-step hybrid process (consisting of evaporation of PbI_2_ and CsBr and subsequent spin-coating of a FAI/MABr/MACl-based solution) was employed to ensure layer conformality.^54^ The final composition is Cs_0.12_FA_0.64_MA_0.24_Pb(I_0.94_Br_0.06_)_3_, achieving a band gap (*E*_*g*_) of 1.63 eV. Likewise, the AZO PBL was revealed to efficiently prevent sputtering damage. The photovoltaic parameters of the opaque and semitransparent two-step hybrid-based devices can be found in the SI. In this case, the tandem device showed a superior *FF* approaching 77% and an efficiency of 25.2% (Figure 5). The photovoltaic parameters are summarized in Table 1. An increased hysteresis was observed, which could be attributed to increased ion migration or to measurement artifacts (i.e., spectral variations in the current mismatch condition).^55^

**Table 1:** Extrapolated Electrical Parameters of the *J–V* Curves in Figure 6A,B, Together with the Parameters for the Standalone SHJ Solar Cell (the *J–V* Curve of SHJ Is Shown in Figure S10)

scan	*V*_OC_ (V)	*J*_SC_ (mA cm^–2^)	fill factor (%)	efficiency (%)	efficiency MPPT (%)
**spin-coated PVK**					
for	1.75	18.05	72.18	22.81	23.2
rev	1.75	17.76	74.58	23.21	
**two-step hybrid PVK**					
for	1.75	18.02	73.49	23.23	25.2
rev	1.76	18.74	76.93	25.31	
**standalone SHJ**					
	0.69	35.40	72.60	17.73	

It is noteworthy that, even though the perovskite band gap in current record perovskite/silicon tandem solar cells is ∼1.67–1.69 eV,^3,16,56^ Aydin *et al.* corroborated that the optimal *E*_*g*_ can vary based on the operating temperature, falling below 1.68 eV under realistic operation conditions.^57^ Therefore, the perovskite band gaps employed here are suitable for the objectives of this research.

The *EQE* measurement disclosed that the SHJ represents the limiting subcell in both two-step hybrid and solution-process cases, with resulting current densities of around 16.3 and 16.5 mA cm^–2^, respectively. The current mismatch is between 1.8 mA cm^–2^ (for the two-step hybrid perovskite based-tandem) and 1.5 mA cm^–2^ (for the solution-processed perovskite based-tandem), with *J*_SC_^PVK^ values equal to 17.6 and 18.03 mA cm^–2^, respectively. This mismatch condition can also be responsible for the high *FF* and could also explain the hysteresis in the two-step hybrid perovskite-based tandem, which might be highly affected by charge accumulation at the recombination contact.^58,59^ The individual simulated absorbance contributions of the layers forming the tandem stack (Figure S12) highlight a significant parasitic absorption within the PCBM layer, primarily in the UV–visible spectrum. The ITO top electrode also consistently contributes to the total absorption across the spectrum, whereas the recombination layer exhibits a high absorption in the near-infrared range. On the other hand, the AZO-based PBL presents a low parasitic absorption, which is approximately 0.2 mA cm^–2^.

**Figure 6:**
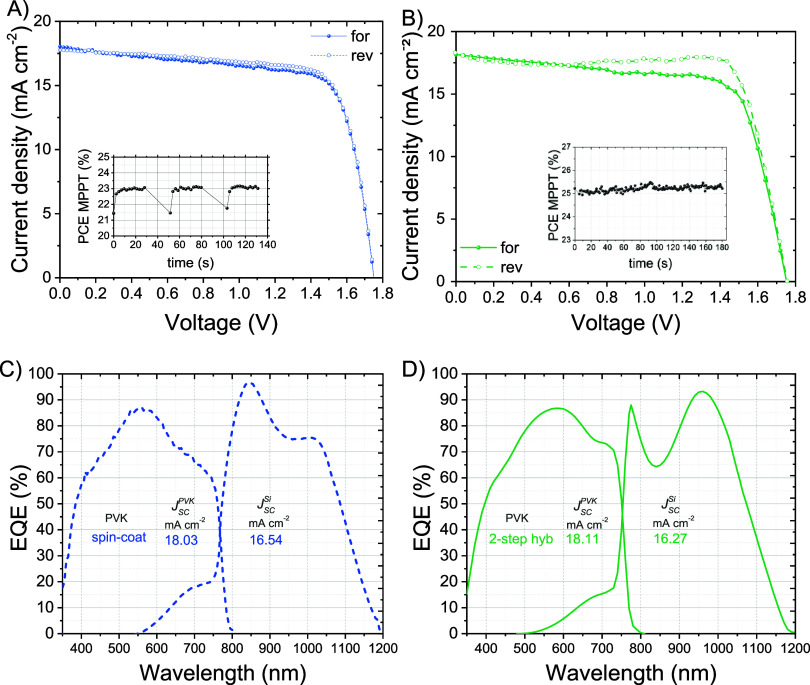
*J–V* curves of the best monolithic perovskite/silicon tandem solar cells based on (A) fully solution processed perovskite top cell and (B) ù two-step hybrid deposition of perovskite. The respective MPPT is shown in the insets. (Discontinuities in MPPT are associated with the algorithm, which alternates between *J–V* curve measurements and MPPT.) The *EQE* spectra of the spin-coated perovskite-based tandem (blue curve, denoted as “spin-coat”) and two-step hybrid-based tandem (green curve, denoted as “2-step hyb”) are reported in (C) and (D), respectively, with the relative integrated current densities.

**Figure 7:**
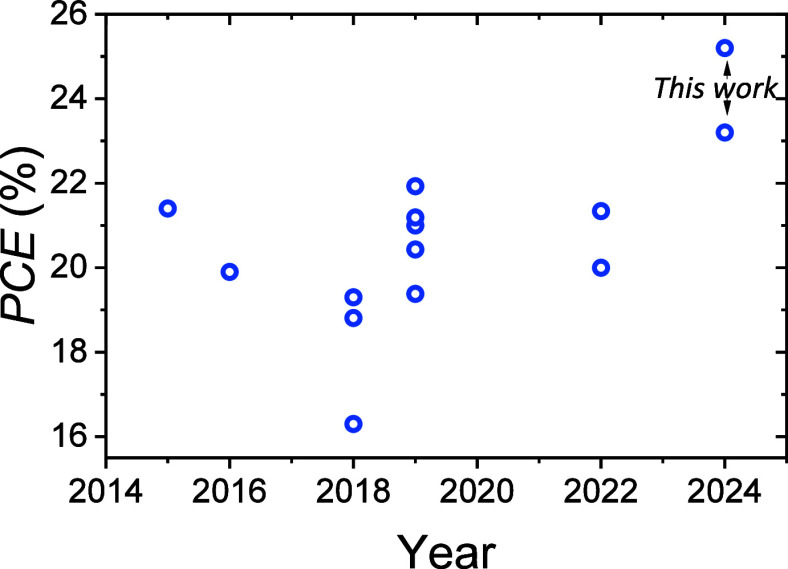
Recent progress on perovskite/silicon tandem solar cells based on fully flat silicon bottom cells from different works.^59−66^ The *PCE*s of our work based on perovskite/silicon tandem with unpolished rear-side/polished front side silicon are also reported in order to highlight the gain obtained from the unpolishing on the silicon backside.

Considering the low σ_RMS_ with respect to the standard texture and the absence of any intentional process for inducing such roughness, it is meaningful to compare our results with tandem devices based on a fully flat silicon bottom cell (Figure 7 and Figure S14).

In this context, we achieved a superior current density by employing a silicon wafer with an unpolished rear-side, taking advantage of improved absorption due to the presence of the back-side roughness and leading to superior performance, as shown in Figure 6.

Moreover, the achieved efficiency is in good agreement with other works using SHJs based on *p*-type silicon wafers (Table S5), even though all these works employ ALD-SnO_2_ as PBL. This agreement strengthens the proposed approach that uses a PBL deposited by a simple spin-coating process and unpolished silicon wafers at the back side for the fabrication of perovskite/silicon tandem cells.

## Conclusions

This work presents a comprehensive investigation focused on optimizing and understanding the crucial role of a solution-processed PBL to achieve efficient ITO-sputtered-based ST-PSCs.

By comparing ZnO, AZO, and SnO_2_ nanoparticle-based solutions, we demonstrated that the AZO solution-processed layer can effectively mitigate sputtering-induced damage, offering a scalable alternative to ALD. The dynamic spin-coating deposition method was identified as a key factor in improving the uniformity and performance of the PBLs, as demonstrated by reduced pinhole defects, as well as improved uniformity and *PCE*s. Among the tested materials, the AZO N-21X-based PBL exhibited superior performance, particularly in terms of resilience to sputtering damage, crystallinity, and UV-shielding property, achieving a *PCE* of 18.1% ST-PSCs. This performance closely matched the best opaque reference devices, confirming the effectiveness of the solution-processed AZO layer. When applied to monolithic perovskite/silicon tandem solar cells, this PBL enabled an efficiency of 23.2% with a fully solution-processed perovskite top cell and 25.3% when using a two-step hybrid perovskite.

A significant outcome of this research was the use of single-side polished *p*-type silicon wafers with an unpolished rear surface. Experiment and simulation confirmed that the native roughness (σ_RMS_ = 241 nm) at the rear surface represents an advantageous trade-off to bypass the need for further texturing processes while still achieving effective light trapping. Considering both the wafer doping type and the absence of intentional texturing processes, this approach holds promise for cost reductions.

This study highlights the potential of solution-processed PBLs as a cost-effective and scalable alternative to ALD in the fabrication of high-efficiency tandem solar cells. By reducing capital expenditure (CapEx) and simplifying processing steps, this approach paves the way for the industrial-scale production of perovskite/silicon tandem cells, offering a significant step toward the commercialization of these high-performance photovoltaic devices.

## Experimental Section

### Materials

Indium–tin oxide (ITO)-coated glasses (*R*_SH_ = 7 Ω *sq*^–1^) were purchased from Kintec. Formamidinium iodide (FAI), methylammonium bromide (MABr), methylammonium chloride (MACl), and PEACl were purchased from GreatCell Solar. Lead(II) iodide (PbI_2_), lead(II) bromide (PbBr_2_), and cesium iodide (CsI) were purchased from TCI. Cesium bromide beads (CsBr), bathocuproine (BCP) and copper beads (Cu beads), ethanol (EtOH) (anhydrous, ≥ 99.8%), acetone (≥99.5%), N,N-Dimethylformamide (DMF) (≥99%), dimethyl sulfoxide (DMSO) (>99%), chlorobenzene (CB) (99.8%), 1,2-dichlorobenzene (DCB) (99%), toluene (>99.7%), 2-propanol (IPA) (anhydrous, 99.5%), and poly(9,9-bis(3′-(*N*,*N*-dimethyl)-*N*-ethylammoinium-propyl-2,7-fluorene)-*alt*-2,7-(9,9-dioctylfluorene))dibromide (PFN-Br), were purchased from Sigma-Aldrich. [6,6]-Phenyl-C_61_-butyric acid methyl ester was purchased from Solenne. ZnO (N-10, N-10-Flex, N-11, N-12), Al:ZnO (3.15 mol % Al) (N-20X-Flex, N-21X, N-21X-Flex), and SnO_2_ (N-30) nanoparticle solutions were purchased from Avantama. Silver (Ag) paste 7713 was purchased from Dupont. Indium-doped Tin Oxide (ITO) target with an In_2_O_3_/SnO_2_ composition of 90:10 wt % for sputtering was purchased from TestBourne Ltd.

### Methods

#### Perovskite Single-Junction Fabrication

The perovskite solar cell has an inverted (*p-i-n*) planar structure. For opaque and semitransparent perovskite solar cells, the glass/ITO sheets were patterned with a Nd:YVO_4_-pulsed UV laser system (BrightSolutions, Luce 40 laser) and then cut into 2.5 × 2.5 cm^2^ samples. The ITO-patterned samples were cleaned in an ultrasonic bath with a 2% solution of Hellmanex detergent in deionized water, acetone, and then isopropanol for 15 min. Any remaining solvent residual was blown off using an air flow. UV-ozone treatment was then performed on the substrates for 15 min to remove all the residual organic contaminants with a PSD Pro Series Digital UV Ozone System from “Novascan”. The samples were then transferred to a nitrogen-filled glovebox and the PTAA (2 mg mL^–1^ in toluene) was spin-coated at 5000 rpm for 20 s. The samples were annealed at 100 °C for 10 min. For PFN-Br deposition, the substrates were spun with a solution (0.5 mg/mL DMF) at 5000 rpm for 20 s, followed by annealing the substrate to 100 °C for 10 min.

After the samples cooled, the perovskite absorber was deposited on the samples. For the fully solution-processed PSCs, the perovskite fabrication can be found elsewhere.^43^ As regards the devices based on two-step hybrid perovskite deposition, a film of PbI_2_ and CsBr (with a ratio of 10:1) was thermally coevaporated onto the substrates. FAI (0.48 M), MABr (0.09 M), and MACl (0.09 M) were dissolved in EtOH and the solution was dynamically spin-coated on the substrates in a flow box filled with dry air (relative humidity, RH < 10%). The samples were then annealed in air (RH between 30 and 40%) at 150 °C for 15 min. On top of the two-step hybrid perovskite layer, PEACl (1.5 mg mL^–1^ in EtOH) was dynamically spin-coated at 4000 rpm for 25 s and subsequently annealed at 100 °C for 10 min. PCBM (27 mg mL^–1^ in CB:DCB, 3:1 volume ratio) was spun at 1350 rpm for 20 s and annealed at 100 °C for 5 min. In the case of the opaque samples, BCP (0.5 mg mL^–1^ in IPA) was deposited at 2300 rpm for 20 s without any further drying. Finally, a 100 nm Cu layer was thermally evaporated on top of the samples using a shadow mask. Conversely, for ST-PSCs fabrication, the PBL solution (ZnO, AZO, or SnO_2_) was sonicated for 15 min prior to use and then statically or dynamically (as stated in the Results and Discussion section) spin-coated in the glovebox on top of PCBM at 3000 rpm for 30 s. Afterward, a rapid annealing of 5 min at 115 °C was performed. A 100 nm-thick ITO layer was then deposited with a linear radio frequency (RF) sputtering system from Kenosistec. A base pressure of 5 × 10^–6^ mbar was reached before starting a presputtering step in order to remove target impurities and to improve the reproducibility of the process. The ITO deposition was carried out with a working pressure of 1.1 × 10^–3^ mbar, a precursor argon flow of 40 sccm, and a power density of 0.39 W cm^–2^. A horizontal motion of the substrate holder was performed to increase the film uniformity. The sheet resistance of the final film is ∼30 Ω/□. Afterward, a copper frame of 100 nm was thermally evaporated using a shadow mask, defining an active area of 0.09 cm^2^.

#### Silicon Cell Preparation

Single-side polished 4 in. *p*-type float-zone Si ⟨100⟩ wafers with resistivity 1–5 Ω cm and thickness 270 μm were employed for SHJ solar cell fabrication. The wafers were cleaned with the RCA procedure and dipped in 2% HF to remove the native oxide. A 5 nm thick intrinsic amorphous silicon passivation layer was deposited on both sides, followed by a *n*-type doped nanocrystalline SiO_*x*_ layer on the polished front side and a *p*-type doped nanocrystalline silicon layer on the unpolished rear side. The Si- and SiO_*x*_-based layers were grown by plasma-enhanced chemical vapor deposition (PECVD) in an MVSystems Inc. cluster tool system. Details on the deposition conditions can be found elsewhere.^52^

Reference single-junction solar cells were fabricated by applying a sputtered AZO/Ag stack as rear contact and an 80 nm-thick antireflective AZO layer followed by a 5 μm-thick Al collection grid, deposited via e-beam evaporation, at the front side. A cell area of 2 × 2 cm^2^ was defined by depositing the TCO layers through shadow masks.

For application in tandems, a 20 nm-thick ITO layer was sputtered at the front side through a shadow mask with 0.36 cm^2^ area openings aligned to AZO/Ag pads with the same area on the rear side. The wafers were finally cut into 2.5 × 2.5 cm^2^ substrates, with each containing four contact pads.

#### Perovskite/Silicon Tandem Solar Cell Fabrication

The silicon substrates were cleaned by spin coating 2-propanol in the glovebox or in ambient air. 150 μL of 2-propanol was dripped statically ∼5 s before starting the spin-coating program at 3000 rpm for 30 s, followed by 150 μL dynamically dropped every ∼10 s. The bottom cells were blown clean with compressed air and UV–O_3_-treated for 15 min. The same HTL, perovskite, (PEACl where stated), PCBM, and AZO N-21X deposition as described above was conducted on the silicon bottom cells. Subsequently, 100 nm ITO was deposited by RF sputtering. A 100 nm copper frame was thermally evaporated through a shadow mask to collect the charge carriers with a central finger. Lastly, a 100 nm MgF_2_ layer was deposited as an antireflective coating by thermal evaporation. The active area is defined by the metal frame and is 0.32 cm^2^. A schematic of the design with four tandem solar cells defined on the substrate is shown in Figure S1.

#### Characterization

*J*–*V* measurements of the PSCs were performed with a Class-A sun simulator (ABET 2000) equipped with an AM1.5G filter (ABET). The calibration of the Sun Simulator was made by using a Si-based reference cell (RR-226-O, RERA Solutions) to obtain a 1 sun illumination condition. Arkeo platform (Cicci Research s.r.l.) was used for *J*–*V* characterization under forward and reverse scan directions and for MPPT. A voltage step of 20 mV/s and a scan rate of 200 mV/s were set.

External quantum efficiency (*EQE*) characterization for single-junction devices was performed with an Arkeo system (Cicci Research s.r.l.) with a 150 W xenon lamp and a double grating (300 to 1400 nm). A Si photodiode was used for incident light calibration prior to the *EQE* measurement.

*EQE* of the tandem devices was measured with a Bentham PVE300 setup.^67^ The *EQE* spectra were recorded between 300 and 1200 nm in 10 nm steps using chopped (133 Hz) monochromatic light from Xe and He lamps. An additional halogen lamp with optical filters was used for light bias. To measure the *EQE* of the perovskite subcell, the silicon subcell was saturated by using red/NIR light bias by applying a long pass filter (Schott RG630). The silicon subcell was measured by saturating the perovskite subcell with blue light using a blue band-pass filter (Schott BG23), together with a coated hot mirror to reflect the IR light. The latter is used to avoid damage to the absorptive blue filter. A correction was applied to the *EQE* spectra, as reported in Figure S11.

Atomic force microscopy (AFM) measurements were performed with the microscope working in the repulsive regime of contact mode in air at room temperature. The Bruker silicon nitride MSNL-10 cantilevers were employed. Constant force images with a force of 1 nN were acquired with a typical scan rate of 2–4 s/row. The data were then analyzed by using Gwyddion software. The scanning electron microscopy (SEM) cross-sectional images were acquired by using field emission SEM (FESEM, Tescan Mira 3 LMU FEG). The thickness measurements were acquired with a Dektak Veeco profilometer.

Photoluminescence (PL) spectroscopy was conducted at room temperature by using a continuous wave laser at 405 nm with an irradiance of 1 W/cm^2^, provided by the Matchbox 2 laser series. The laser beam was focused onto the sample with a spot size of 100 μm, and the emitted PL was collected by a telescope and directed into a monochromator (HORIBA Jobin Yvon–iHR320) with a 320 mm focal length. The monochromator had a spectral range of 350 to 1100 nm and was equipped with a 1200 gr/mm grating.

X-ray diffraction (XRD) measurements were performed with a Rigaku SmartLab diffractometer, working in (ϑ—2ϑ) Bragg–Brentano geometry, equipped with a Cu source (Kα_1_ = 1.54056 Å, Kα_2_ = 1.54439 Å) and a D/teX Ultra 250 silicon strips detector. XRD spectra were collected over a 20° to 80° 2θ range in a single scan, with a step size of 0.01° and a scan speed of 8°/min.

A homemade Kelvin Probe system was employed for work function measurement. The apparatus is equipped with a piezoelectric-driven probe with a 2 × 1.5 mm gold mesh serving as a reference electrode.

#### Optoelectronic Model and Simulations

Optoelectronic simulations were performed by using the Setfos Software.^53^ The employed optical parameters, i.e., refractive indexes (*n*) and extinction coefficient (*k*), are reported in Figure S13 and are either obtained by spectral ellipsometry measurements (PTAA) or taken from the literature.^68,69^ The perovskite top subcell consists of a 30 nm PTAA layer, 450 nm perovskite layer, 60 nm PCBM, 20 nm AZO buffer layer, and 100 nm ITO electrode. The employed thicknesses of the silicon subcell are reported in Figure S8A, with 20 nm of ITO as the recombination layer. The cell architecture is completed by 80 nm of a MgF_2_ antireflective coating. The thin-film layers were considered as conformally deposited on top/bottom of the 280 μm-thick silicon layer. Thus, the optical behavior of the coherent stack was computed by the transfer matrix method and then used as input for the 3D ray tracer, which accounts for scattering induced by rough interfaces, if any. For this purpose, data on silicon substrate roughness based on AFM measurements (Figure S8E) were used. This approach was also used when a regular pyramid texture was assumed for the silicon rear side. In this case, a 3D texture map, shown in Figure S8F, was generated within the Setfos suite. The computed optical generation profile was used as input for transport simulations.^68^

## Supporting Information Available

Material details of nanoparticle dispersions; *J–V* characteristics and *EQE* spectra; optoelectronic simulations; AFM measurements and 3D maps; SEM; PL; absorptance spectra (PDF)
