# Supplementary material for: Solution-Processed Metal-Oxide Nanoparticles to Prevent The Sputtering Damage in Perovskite/Silicon Tandem Solar Cells
Source: ACS Appl Mater Interfaces. 2025 Mar 10;17(11):17599–610. doi: 10.1021/acsami.5c00090 (PMC11931493; doi:10.1021/acsami.5c00090)
Supplement: Supplementary file 1 — am5c00090_si_001.pdf [file am5c00090_si_001.pdf]

Supporting Information

Solution-Processed Metal-Oxide Nanoparticles To Prevent The Sputtering Damage In Perovskite/Silicon Tandem Solar Cells

Erica Magliano[a], Francesco Di Giacomo[a$], Harshavardhan Reddy Sathy[a#], Shirin M. Pourmotlagh[a£], Gemma Giliberti[b], David Becerril Rodriguez[c], Giuseppe Ammirati[c], Paolo Mariani[a], Francesca Zarotti[a], Fabio Matteocci[a], Marco Luce[c], Iurie Usatii[d], Eugenia Bobeico[d], Marco Della Noce[d], Antonio Cricenti[c], Federica Cappelluti[b], Lucia V. Mercaldo[d], Paola Delli Veneri[d], and Aldo Di Carlo[a,c*]*

[a] C.H.O.S.E. (Center for Hybrid and Organic Solar Energy), Electronic Engineering Department, University of Rome Tor Vergata, Via del Politecnico 1, Rome, 00118, Italy.

[b] Department of Electronics and Telecommunication, Politecnico di Torino, Corso Duca degli Abruzzi 24, Turin, 10129, Italy.

[c] Istituto di Struttura della Materia (CNR-ISM) National Research Council, via del Fosso del Cavaliere 100, Rome, 00133, Italy.

[d] ENEA – Portici Research Center, P.le Enrico Fermi 1, Portici (Naples), 80055, Italy.

* Email: aldo.dicarlo@uniroma2.it

* Email: lucia.mercaldo@enea.it

Material	*SnO_2*	*Al:ZnO (3.15 mol% Al)*			*ZnO*			
Code Name	N-30	N-21X-Flex	N-21X	N-20X-Flex	N-12	N-11	N-10-Flex	N-10
Particle Size (nm)	7	12	12	12	12	12	12	12
Hydrodynamic particle size (nm)	20	18	18	18	12	19	15	16
Work Function (eV)	4.1	3.9	3.9	4.3	4.3	3.9	4.3	4.3
Concentration (Wt%)	2.5	2.5	2.5	2.5	5	2.5	2.5	2.5
Solvent	Mixture of butanols	Mixture of butanols	Mixture of alcohols	Mixture of butanols	Ethanol	Mixture of alcohols	Mixture of butanols	2-propanol
Viscosity (CP)	3.5	3	2.2	3	1.2	2.1	2.8	2.4
Coating Applicability	Spin coating Blade coating Slot-die coating	Spin coating Blade coating Slot-die coating	Spin coating Blade coating Slot-die coating	Spin coating Blade coating Slot-die coating	Spin coating Blade coating	Spin coating Blade coating Slot-die coating	Spin coating Blade coating Slot-die coating	Spin coating Blade coating Slot-die coating

Table S1: A comprehensive overview of the buffer layers employed in the present investigation, providing detailed information for each metal-oxide ink from Avantama.

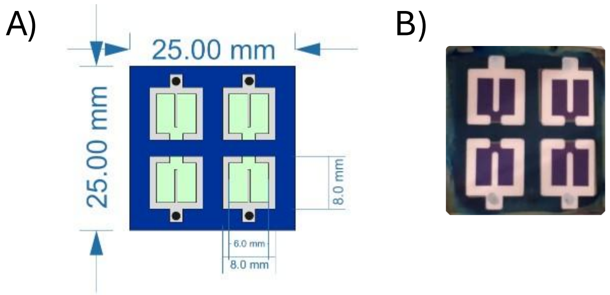

Figure S1: A) Layout of the samples with four tandem cells per substrate. B) Picture of a standard tandem device.

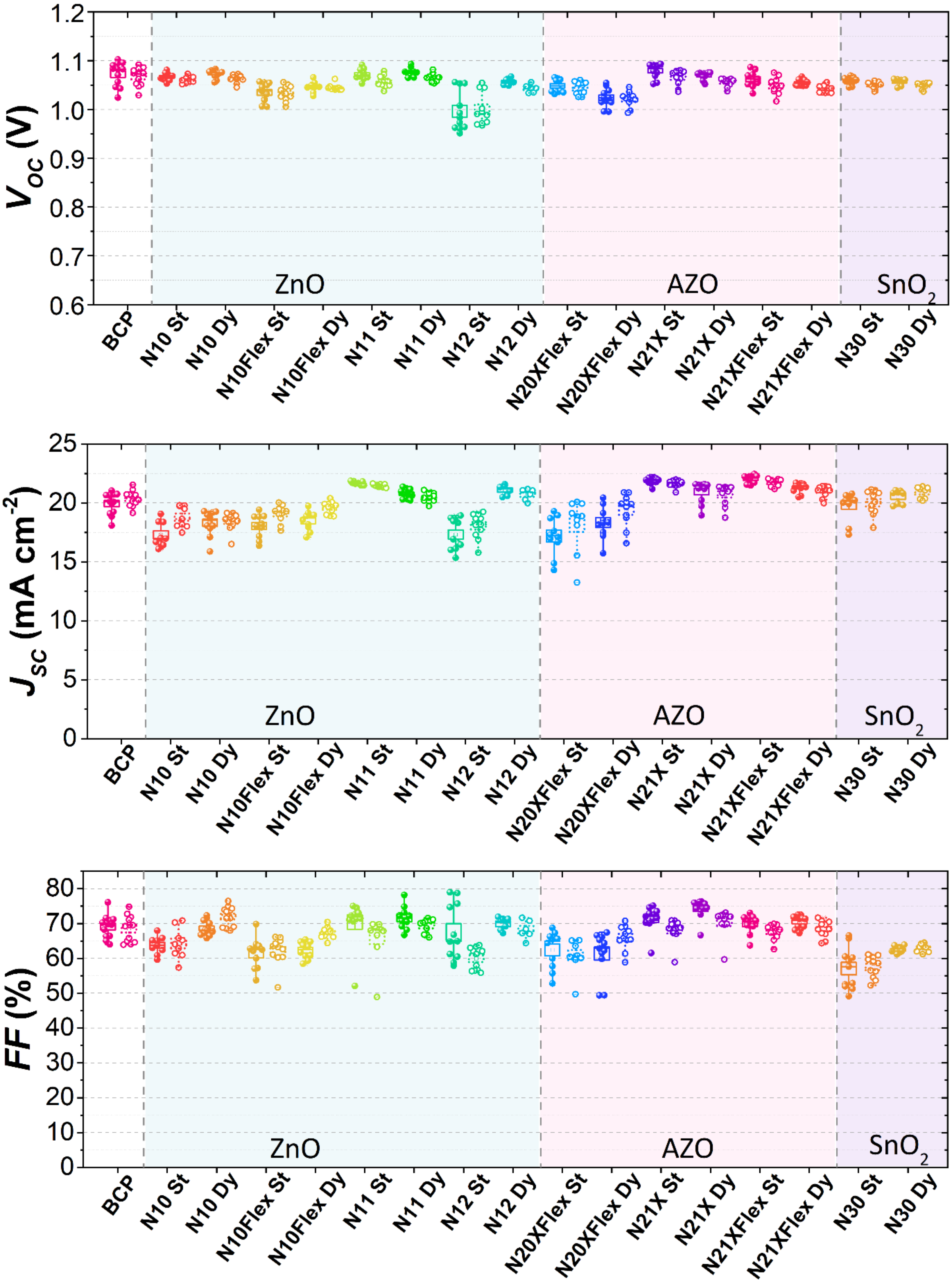

Figure S2: Statistical distribution of V_{OC}, J_{SC}, FF in reverse (closed dot) and forward (open dots) scan of perovskite solar cells (glass/ITO/PTAA/PFN-Br/$Cs_{0.05}MA_{0.14}FA_{0.81}PbI_{2.7}Br_{0.3}$/$PC_{61}BM$/PBL/Cu) with various solution-processed PBLs deposited via static (St) and dynamic (Dy) coating.

PBL		V_{OC} V	J_{SC} mA cm^{-2}	*FF* %	*PCE* %
BCP		1.07±0.02 (1.1)	19.94±0.88 (21.05)	69.14±3.2 (76.07)	14.83±1.41 (17.38)
ZnO	N-10 (Static)	1.07±0.01 (1.08)	17.3±0.99 (19.07)	63.73±2.44 (67.92)	11.77±0.85 (13.25)
	N-10 (Dynamic)	1.07±0.01 (1.08)	18.32±0.98 (19.3)	68.62±2.02 (72.3)	13.5±0.85 (14.56)
	N-10-Flex (Static)	1.04±0.02 (1.06)	18.04±0.91 (19.42)	61.48±4.14 (69.83)	11.46±0.72 (12.59)
	N-10-Flex (Dynamic)	1.05±0.01 (1.07)	18.5±0.77 (19.75)	62.32±2.31 (65.35)	12.06±0.48 (12.78)
	N-11 (Static)	1.07±0.01 (1.09)	21.69±0.13 (21.88)	70.41±6.26 (75.02)	16.36±1.42 (17.34)
	N-11 (Dynamic)	1.08±0.01 (1.09)	20.78±0.39 (21.38)	71.61±3.16 (78.17)	16.02±0.79 (17.16)
	N-12 (Static)	1.00±0.04 (1.06)	17.31±1.24 (18.94)	67.54±7.68 (78.95)	11.55±0.56 (12.48)
	N-12 (Dynamic)	1.06±0.01 (1.07)	21.1±0.42 (21.59)	69.85±1.76 (71.89)	15.58±0.74 (16.44)
AZO	N-20X-Flex (Static)	1.05±0.01 (1.07)	17.19±1.55 (19.3)	62.39±5.16 (68.72)	11.22±1.13 (12.19)
	N-20X-Flex (Dynamic)	1.02±0.02 (1.05)	18.87±2.09 (22.23)	61.36±6.05 (67.41)	11.78±1.04 (12.87)
	N-21X (Static)	1.08±0.01 (1.09)	21.88±0.28 (22.2)	71.41±3.48 (75.1)	16.85±0.74 (17.58)
	N-21X (Dynamic)	1.07±0.01 (1.08)	20.97±0.86 (21.6)	74.33±2.6 (76.28)	16.67±0.93 (17.55)
	N-21X-Flex (Static)	1.06±0.01 (1.09)	22.02±0.32 (22.46)	69.85±2.54 (73.01)	16.33±0.62 (17.06)
	N-21X-Flex (Dynamic)	1.06±0.01 (1.07)	21.31±0.38 (21.71)	70.42±1.77 (72.46)	15.84±0.64 (16.46)
SnO_2	N-30 (Static)	1.06±0.01 (1.07)	19.81±1.09 (20.78)	57.05±5.65 (66.55)	11.9±0.73 (12.93)
	N-30 (Dynamic)	1.06±0.01 (1.06)	20.52±0.54 (21.08)	62.46±0.91 (63.9)	13.54±0.28 (13.87)

Table S2: Electrical parameters (V_{OC}, J_{SC}, FF, PCE.) in reverse scan of the different types of opaque devices with BCP, ZnO, AZO, or SnO_x as buffer layer. The mean and standard deviation values are indicated, and the maximum measured value is shown in the brackets.

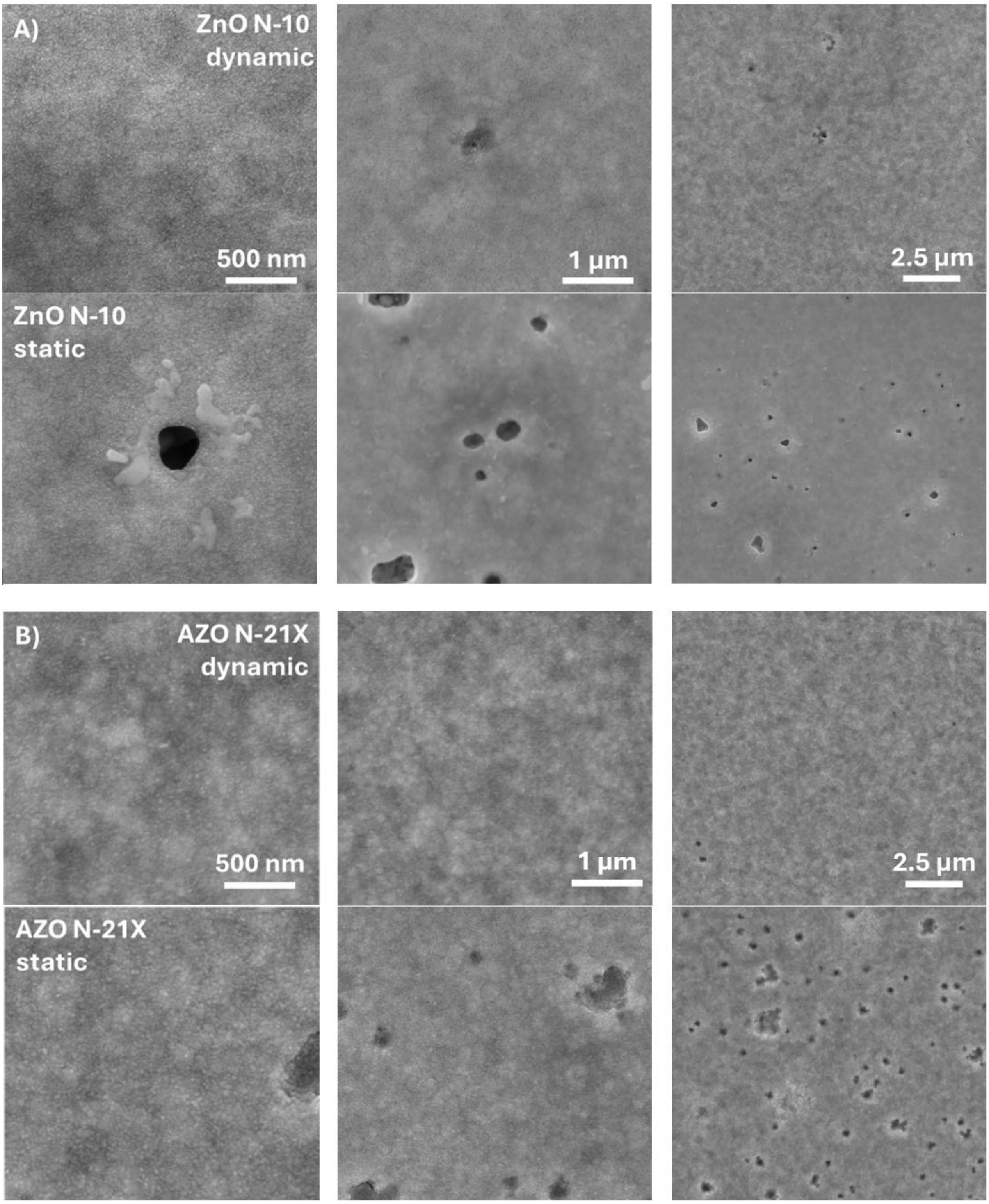

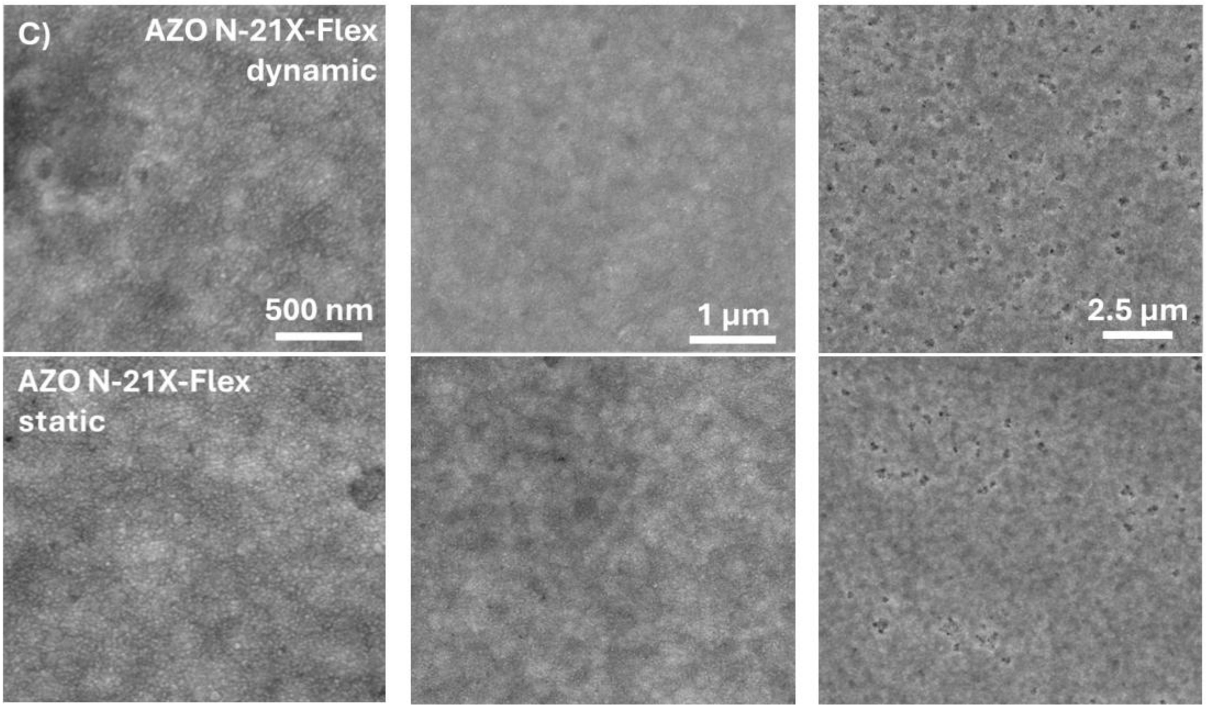

Figure S3: SEM images of glass/ITO/PTAA/PFN-Br/perovskite/PCBM and A) ZnO, B) AZO N-21X, or C) AZO N-21X-Flex on top, with the PBL deposited dynamically or statically.

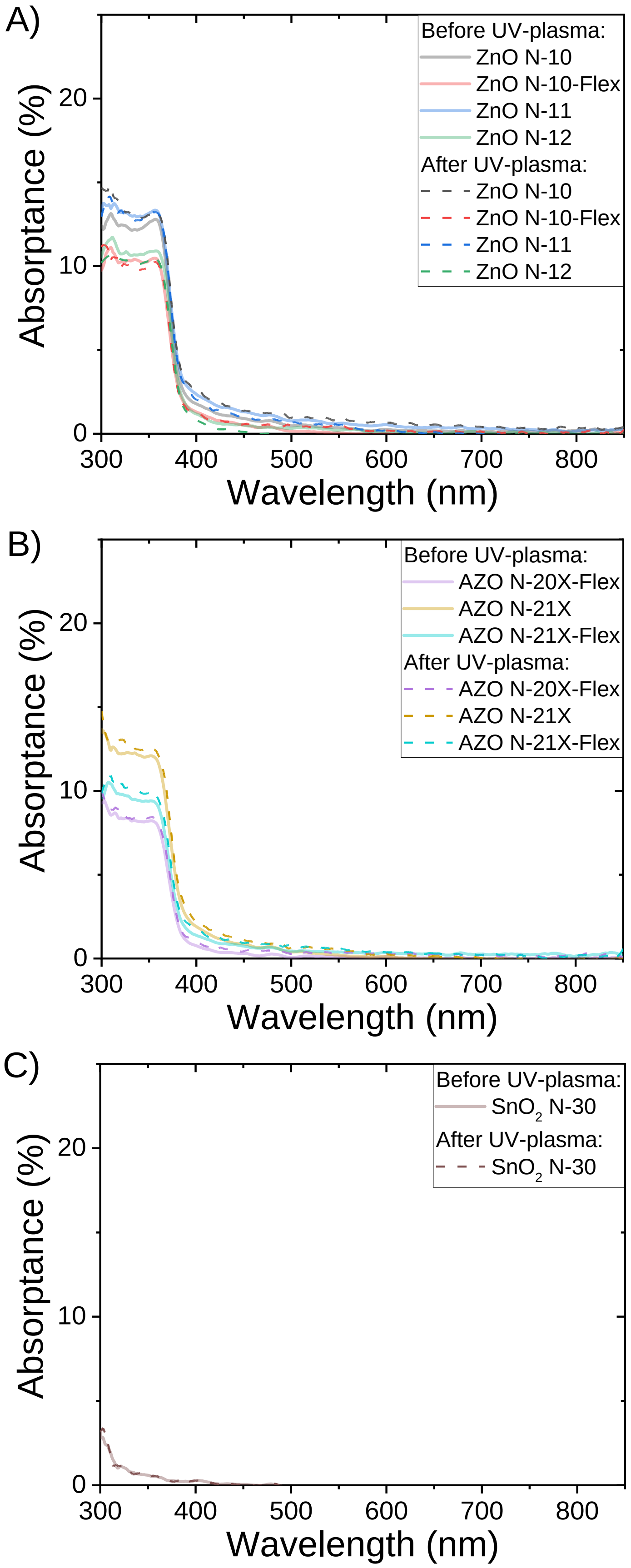

Figure S4: Absorptance spectra of A) ZnO (N-10, N-10-Flex, N-11, N-12), B) AZO (N-20X-Flex, N-21X, N-21X-Flex) and C) SnO_2 (N30) as bare films, before (continuous line) and after (dashed line) plasma exposure during sputtering process.

The simplified rate equation for a PSC in open circuit conditions under illumination is:

$$\frac{dn}{dt} = -An - Bn^2 + g \tag{1}$$

where n is the electronic charge carrier density, A is the first-order Shockley−Read−Hall (SRH) trapping (non-radiative) rate constant (between 1 10^6 and 2.5 10^4 s^{-1}), B is the second-order band-to-band (radiative) recombination rate constant (~ 10^{-10} cm^3 s^{-1}) and g is the generation rate (approximately 10^{22} cm^{-3} s^{-1}).[1], [2]

In steady-state condition, the following is valid:

$$\frac{dn}{dt} = 0 \rightarrow \frac{dn}{dt} = -An - Bn^2 + g \rightarrow n = \frac{A \pm \sqrt{A^2 + 4Bg}}{2B} \tag{2}$$

The photoluminescence quenching (PLQ) before (n_{Ctrl}) and after (n_{UV}) UV-plasma exposure is given by:

$$PLQ = 1 - \frac{n_{UV}^2}{n_{Ctrl}^2} \tag{3}$$

Therefore, by replacing the (2) in (3), we obtain:

$$PLQ \approx 1 - \frac{A_{UV}^2}{A_{Ctrl}^2} \tag{4}$$

This result indicates that the observed PLQ is primarily due to an increase in the non-radiative recombination rate, which arises from the formation of additional non-radiative recombination centers following UV-plasma treatment. An example of PL spectra before (black curve) and after (red curve) UV-plasma exposure is shown in Figure S5.

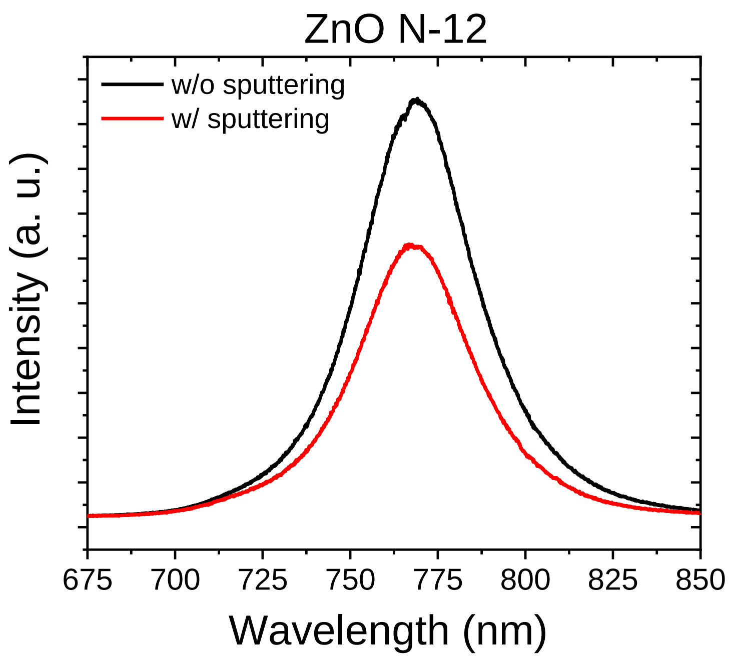

Figure S5: PL spectra of a sample glass/ITO/HTL/Perovskite/PCBM/ZnO N-12 before (black curve) and after (red curve) UV-plasma exposure.

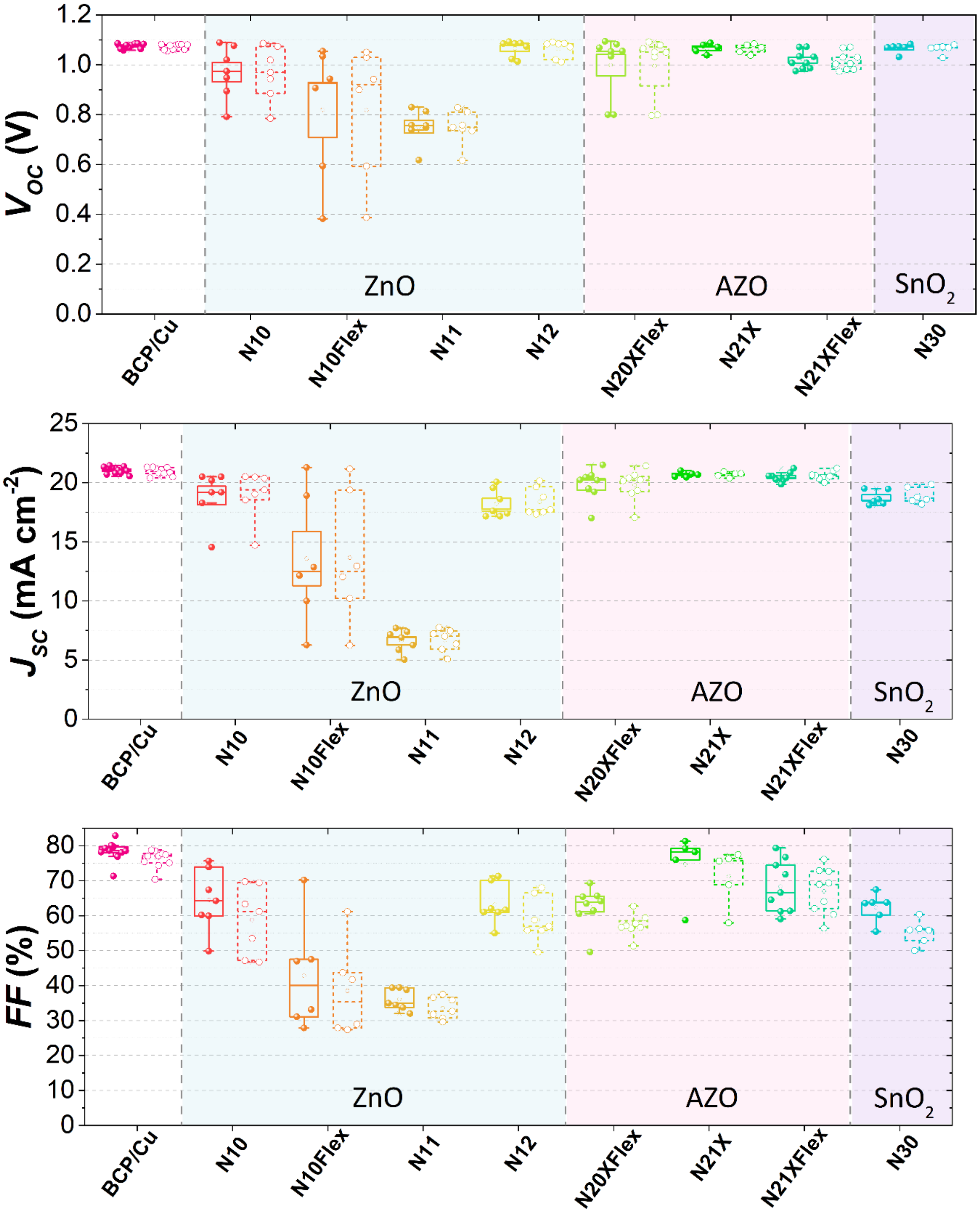

Figure S6: V_{OC}, J_{SC}, FF of semitransparent perovskite solar cells (glass/ITO/PTAA/$Cs_{0.05}MA_{0.14}FA_{0.81}Pb I_{2.7}Br_{0.3}$/$PC_{61}BM$/PBL/ITO) with different solution-processed buffer layers.

PBL		V_{OC}<br>V	J_{SC}<br>mA cm^{-2}	*FF*<br>%	*PCE*<br>%
BCP/Cu		1.07±0.01<br>(1.09)	21.04±0.31<br>(21.46)	78.34±2.95<br>(82.90)	17.73±0.74<br>(18.44)
ZnO	N-10	0.97±0.10<br>(1.09)	18.91±2.09<br>(20.50)	64.46±8.90<br>(75.66)	12.02±3.27<br>(16.65)
	N-10-Flex	0.82±0.27<br>(1.05)	13.58±5.60<br>(21.29)	42.80±15.78<br>(70.21)	5.59±5.01<br>(14.01)
	N-11	0.75±0.07<br>(0.83)	6.62±0.94<br>(7.70)	36.11±3.04<br>(39.45)	1.82±0.43<br>(2.27)
	N-12	1.00±0.12<br>(1.09)	18.22±1.20<br>(20.06)	63.15±5.69<br>(71.32)	12.26±1.40<br>(14.50)
AZO	N-20X-Flex	0.99±0.12<br>(1.09)	19.85±1.34<br>(21.51)	62.47±5.85<br>(69.30)	12.52±2.75<br>(14.91)
	N-21X	1.07±0.02<br>(1.09)	20.68±0.22<br>(21.02)	74.68±9.13<br>(81.27)	16.51±2.30<br>(18.13)
	N-21X-Flex	1.02±0.04<br>(1.07)	20.48±0.39<br>(21.22)	68.34±7.45<br>(79.36)	14.29±1.93<br>(17.50)
SnO_2	N-30	1.07±0.02<br>(1.08)	18.73±0.61<br>(19.50)	62.37±4.09<br>(67.43)	12.47±1.00<br>(14.22)

Table S3: Electrical parameters (V_{OC}, J_{SC}, FF, PCE.) in reverse scan of the different types of opaque device (BCP/Cu) and of ST-PSCs based on ZnO, AZO, or SnO_x as buffer layer. The mean and standard deviation values are indicated, and the maximum measured value is shown in the brackets.

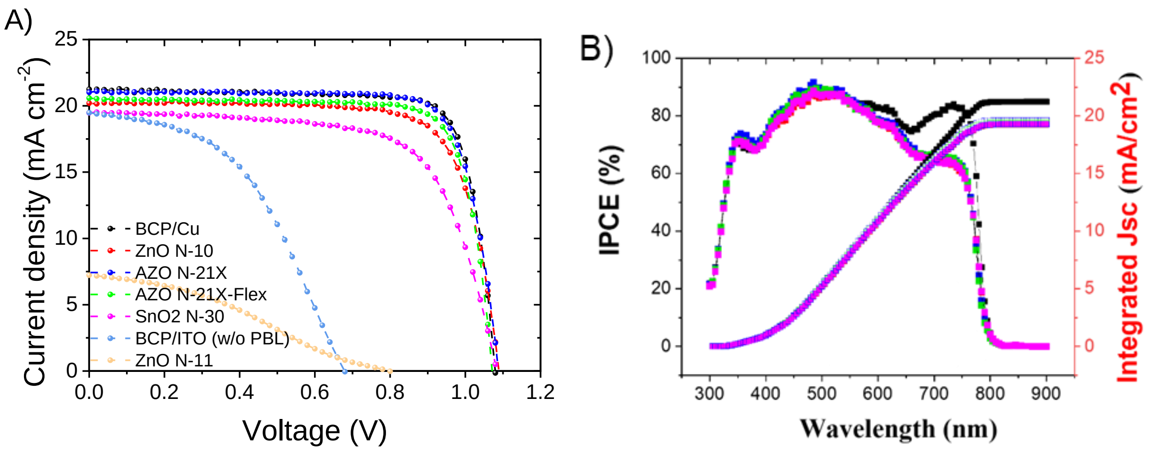

Figure S7: A) Comparison of the J–V curves of the reference opaque device (black) and the ST-PSCs based on the best performing PBLs, as well as a ST-PSC without PBL, and with ZnO N-11, showing the typical S-shape. The relative EQE are reported in (B), whereas the electrical values can be found in Table S4.

Device	***V_{OC}***	***J_{SC}***	***$J_{SC\text{-}EQE}$***	***R_S***	***R//***	***FF***	***PCE***
	**V**	**mA/cm²**	**mA/cm²**	**Ω**	**Ω**	**%**	**%**
BCP/Cu	1.0795	21.24	21.22	6.59E^{+01}	1.59E^{+04}	80.15	18.38
ZnO N-10	1.0894	20.19	19.25	6.94E^{+01}	2.94E^{+03}	75.66	16.65
AZO N-21X	1.0880	21.02	19.62	6.38E^{+01}	1.74E^{+04}	79.25	18.13
AZO N-21X-Flex	1.0714	20.58	19.42	6.28E^{+01}	4.83E^{+03}	79.36	17.5
SnO_2 N-30	1.0837	19.46	19.28	9.47E^{+01}	8.97E^{+03}	67.43	14.22

Table S4: Electrical parameters of the best devices with the PBL/electrode stack indicated in the first column. The J-V curves are shown in Figure S7-A.

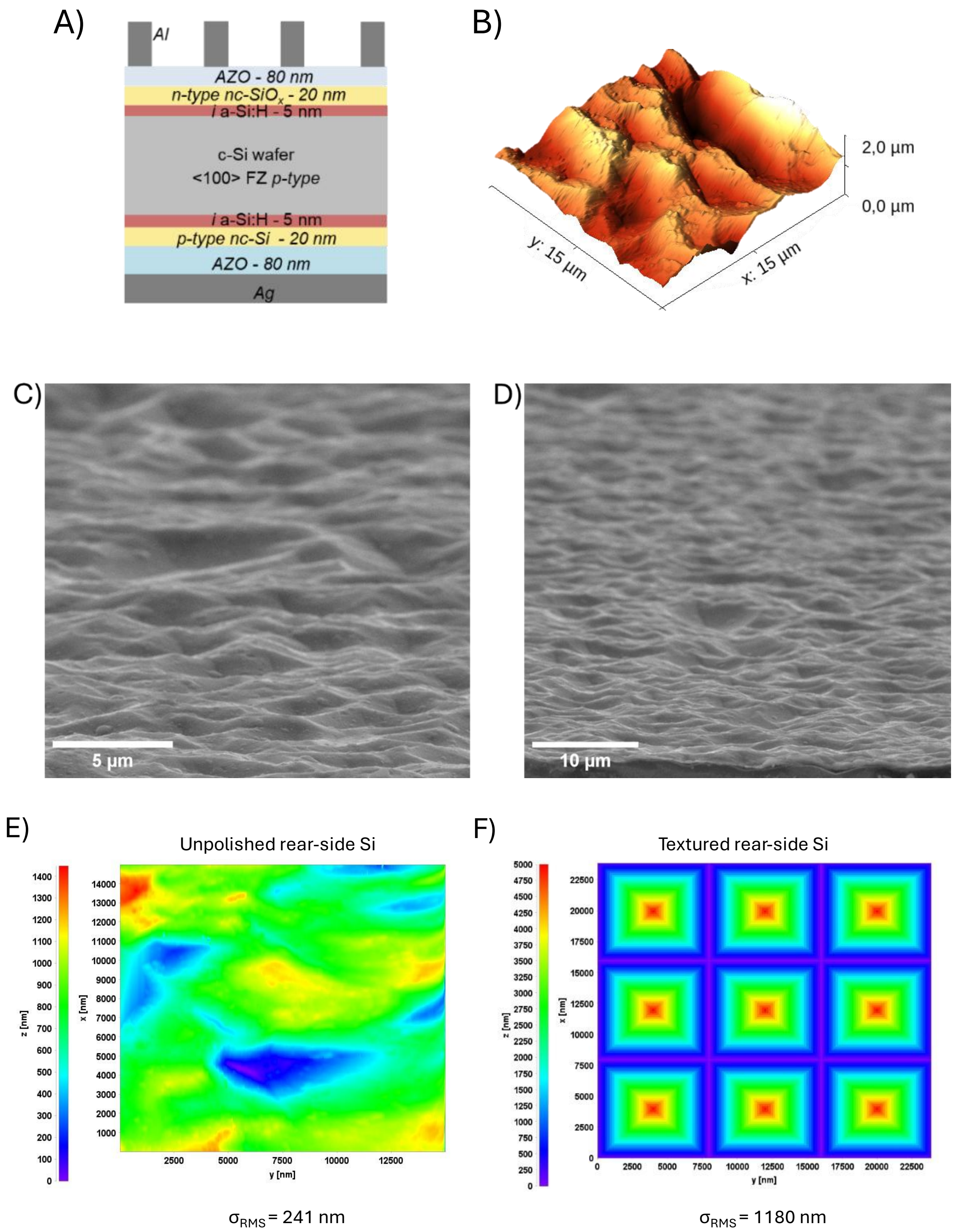

Figure S8: A) Schematics of silicon heterojunction (SHJ) reference cells. B) 3D AFM height image of the unpolished rear side of the SHJ. C, D) Scanning electron microscopy (SEM) images of the unpolished rear side of the SHJ. E) AFM map of the rear surface of an unpolished silicon wafer, imported for optoelectronic simulations (dashed red curve in the EQE plot in the main manuscript). F) 3D map of the simulated periodic texture (dashed blue curve in the EQE plot in the main manuscript).

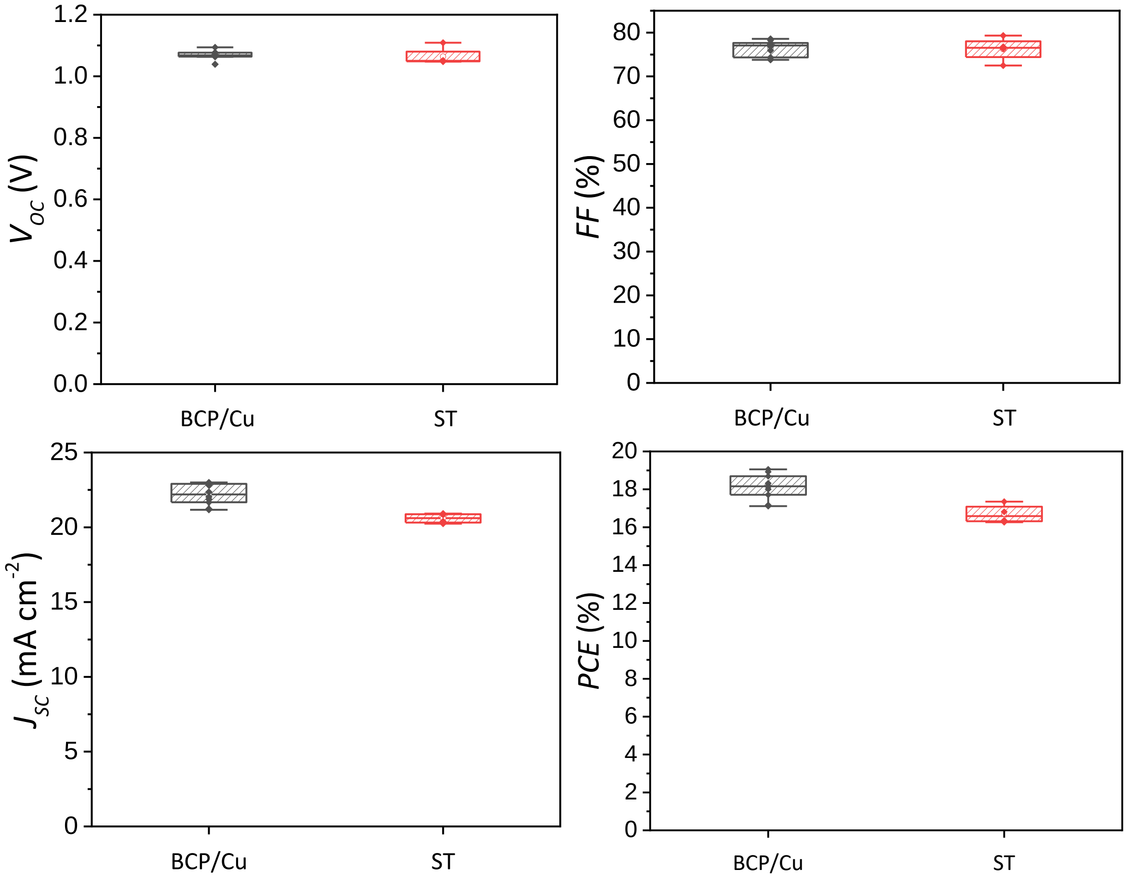

Figure S9: Box plots of the main electrical parameters (V_{OC}, FF, J_{SC}, PCE) relative to the opaque (black plot) and semitransparent (red plot) devices based on two-step hybrid perovskite.

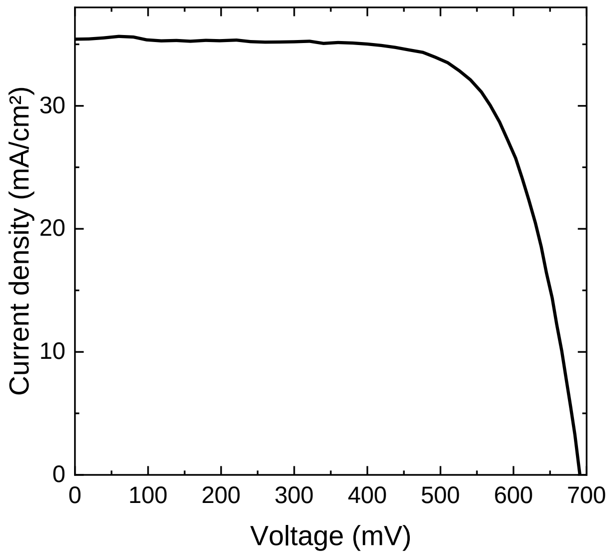

Figure S10: J-V curve of the reference standalone SHJ cell corresponding to the bottom cell used for the fabrication of the tandem solar cells.

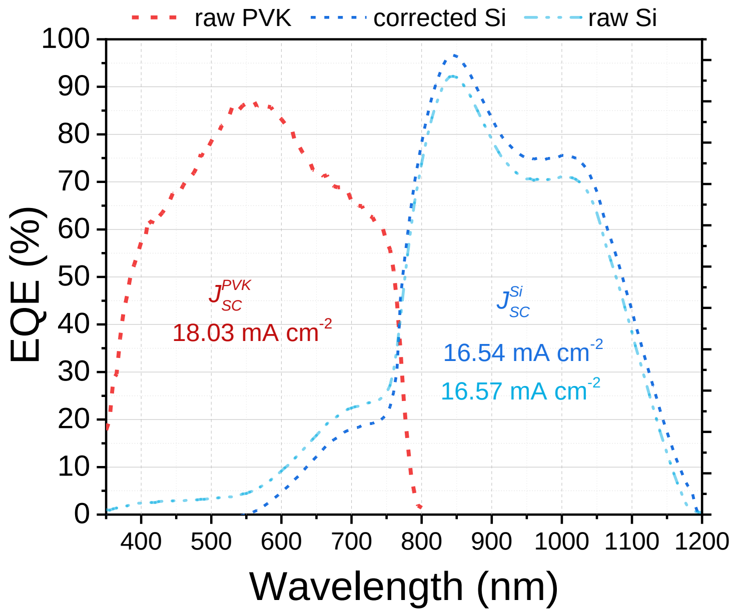

Figure S11: External quantum efficiency (EQE) spectra of the perovskite (red) and silicon (light blue) subcells in the tandem solar cell as obtained from the EQE measurement (raw). In order to account for a measurement artifact on the bottom cell between 300-550 nm, the EQE of the silicon subcell was corrected (dark blue curve) as presented in Siefer G. et al's work.[3] *The respective integrated current densities are reported in the graph.*

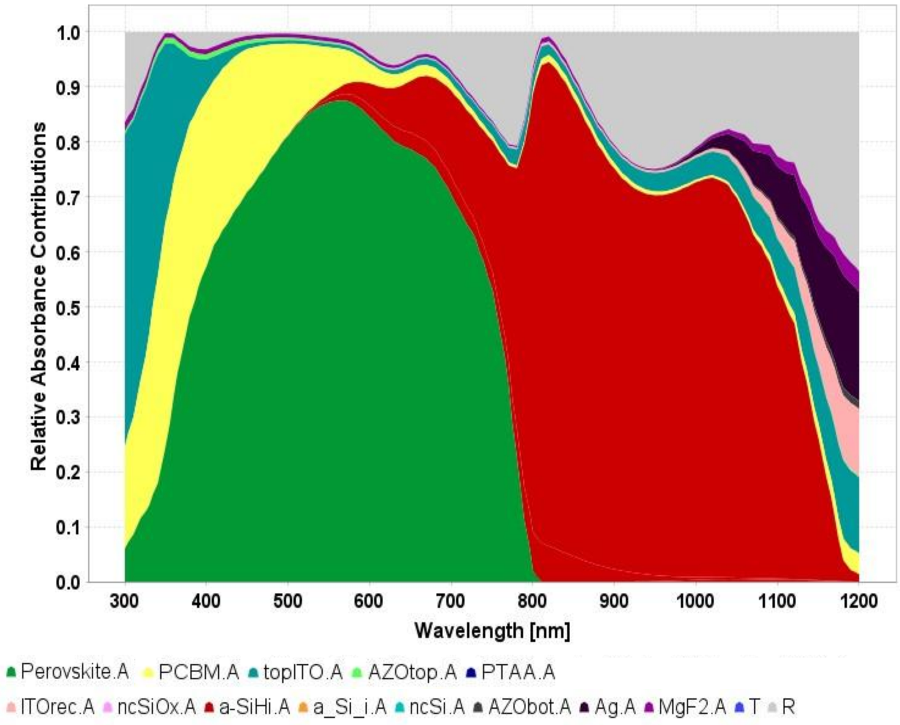

Figure S12: Relative absorbance contributions of the 2T perovskite/silicon tandem stack.

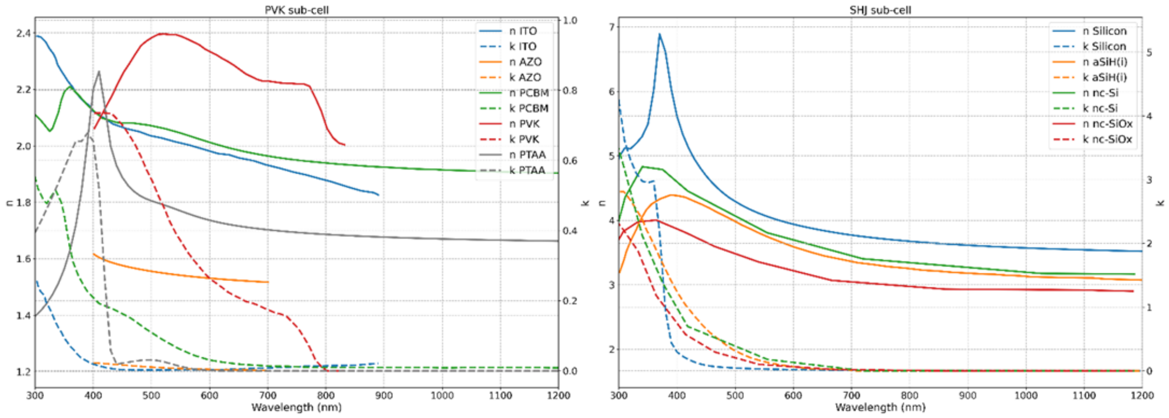

Figure S13: Refractive index (n) and extinction coefficient (k) of each layer for the perovskite top subcell (on the left) and for the SHJ subcell (on the right) employed for the optoelectronic simulations.[4]

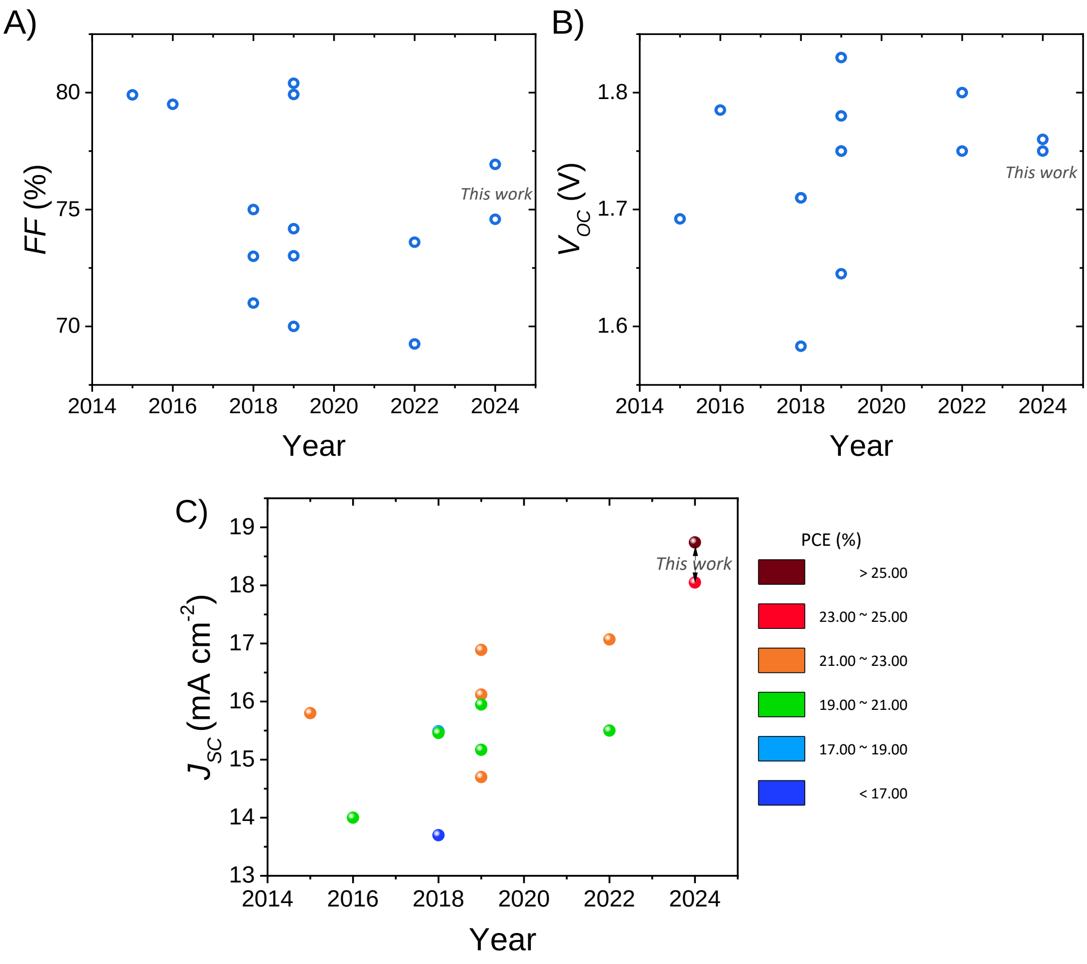

Figure S14: Recent progress on perovskite/silicon tandem solar cells based on fully flat silicon bottom cells from different works ([5]–[12]). The FF and V_{OC} are reported in A) and B), respectively. C) Colormap plot of the current densities of perovskite/silicon tandem solar cells based on fully flat silicon bottom cells from different works ([5]–[12]), over time as a function of the PCE. The PCE is reported in the main text.

**Year**	2019	2020	2023	2023	2024	2024
J_{SC} **mA cm^{-2}**	19.5	17.7	19.3	20.1	20.1	18.7
V_{OC} **V**	1.741	1.77	1.775	1.775	1.903	1.76
FF **%**	74.7	80.3	78.4	74.8	78.6	76.9
PCE **%**	25.4	25.1	26.8	26.6	30.1% (certified: 28.5%)	**25.3**
**Certification**	no	yes	yes	no	yes	no
**Aperture area (cm^2)**	1.42	0.25	0.25	1	1	0.32
**Details**	fully textured p-i-n PVK/SHJ based on p-type wafer	textured rear-side/flat front-side p-i-n PVK/SHJ based on p-type wafer	textured rear-side/flat front-side p-i-n PVK/SHJ based on p-type wafer	fully textured p-i-n PVK/SHJ based on p-type wafer	fully textured p-i-n PVK/SHJ based on p-type wafer	**unpolished rear-side/flat front-side** p-i-n PVK/SHJ based on **p-type** wafer
**Ref.**	[17]	[16]	[15]	[14]	[13]	THIS WORK

Table S5: Electrical parameters, device details and publication information of works on perovskite/silicon tandem solar cells based on p-type SHJ.

REFERENCES

[1] L. M. Herz, "Charge-Carrier Dynamics in Organic-Inorganic Metal Halide Perovskites," *Annu. Rev. Phys. Chem.*, vol. 67, no. 1, pp. 65–89, May 2016, doi: 10.1146/annurev-physchem-040215-112222.

[2] V. Campanari *et al.*, "Reevaluation of Photoluminescence Intensity as an Indicator of Efficiency in Perovskite Solar Cells," *Sol. RRL*, vol. 6, no. 8, Aug. 2022, doi: 10.1002/solr.202200049.

[3] G. Siefer, C. Baur, and A. W. Bett, "External quantum efficiency measurements of Germanium bottom subcells: Measurement artifacts and correction procedures," in *2010 35th IEEE Photovoltaic Specialists Conference*, IEEE, Jun. 2010, pp. 000704–000707. doi: 10.1109/PVSC.2010.5616919.

[4] G. Giliberti, M. Cagnoni, and F. Cappelluti, "Monolithic 3-terminal perovskite/silicon HBT-based tandem compatible with both-side contact silicon cells: a theoretical study," *EPJ Photovoltaics*, vol. 14, p. 37, Nov. 2023, doi: 10.1051/epjpv/2023024.

[5] J. Werner *et al.*, "Efficient Monolithic Perovskite/Silicon Tandem Solar Cell with Cell Area >1 cm 2," *J. Phys. Chem. Lett.*, vol. 7, no. 1, pp. 161–166, Jan. 2016, doi: 10.1021/acs.jpclett.5b02686.

[6] S. Albrecht *et al.*, "Monolithic perovskite/silicon-heterojunction tandem solar cells processed at low temperature," *Energy Environ. Sci.*, vol. 9, no. 1, pp. 81–88, 2016, doi: 10.1039/C5EE02965A.

[7] F. Hou *et al.*, "Inverted pyramidally-textured PDMS antireflective foils for perovskite/silicon tandem solar cells with flat top cell," *Nano Energy*, vol. 56, pp. 234–

240, Feb. 2019, doi: 10.1016/j.nanoen.2018.11.018.

[8] S. Mariotti *et al.*, “Monolithic Perovskite/Silicon Tandem Solar Cells Fabricated Using Industrial p-Type Polycrystalline Silicon on Oxide/Passivated Emitter and Rear Cell Silicon Bottom Cell Technology,” *Sol. RRL*, vol. 6, no. 4, Apr. 2022, doi: 10.1002/solr.202101066.

[9] S. Zhu *et al.*, “Transparent electrode for monolithic perovskite/silicon-heterojunction two-terminal tandem solar cells,” *Nano Energy*, vol. 45, pp. 280–286, Mar. 2018, doi: 10.1016/j.nanoen.2017.12.043.

[10] J. Zheng *et al.*, “Large area efficient interface layer free monolithic perovskite/homo-junction-silicon tandem solar cell with over 20% efficiency,” *Energy Environ. Sci.*, vol. 11, no. 9, pp. 2432–2443, 2018, doi: 10.1039/C8EE00689J.

[11] C. O. Ramírez Quiroz *et al.*, “Interface Molecular Engineering for Laminated Monolithic Perovskite/Silicon Tandem Solar Cells with 80.4% Fill Factor,” *Adv. Funct. Mater.*, vol. 29, no. 40, Oct. 2019, doi: 10.1002/adfm.201901476.

[12] F. Hou *et al.*, “Monolithic Perovskite/Silicon-Heterojunction Tandem Solar Cells with Open-Circuit Voltage of over 1.8 V,” *ACS Appl. Energy Mater.*, vol. 2, no. 1, pp. 243–249, Jan. 2019, doi: 10.1021/acsaem.8b00926.

[13] O. Er-raji *et al.*, “Tailoring perovskite crystallization and interfacial passivation in efficient, fully textured perovskite silicon tandem solar cells,” *Joule*, vol. 8, no. 10, pp. 2811–2833, Oct. 2024, doi: 10.1016/j.joule.2024.06.018.

[14] O. Er-raji *et al.*, “Toward efficient and industrially compatible fully textured perovskite silicon tandem solar cells: Controlled process parameters for reliable perovskite

formation," *Prog. Photovoltaics Res. Appl.*, no. July, pp. 1–14, 2023, doi: 10.1002/pip.3770.

[15] M. Heydarian *et al.*, "Maximizing Current Density in Monolithic Perovskite Silicon Tandem Solar Cells," *Sol. RRL*, vol. 7, no. 7, Apr. 2023, doi: 10.1002/solr.202200930.

[16] P. S. C. Schulze *et al.*, "25.1% High-Efficiency Monolithic Perovskite Silicon Tandem Solar Cell with a High Bandgap Perovskite Absorber," *Sol. RRL*, vol. 4, no. 7, pp. 1–10, 2020, doi: 10.1002/solr.202000152.

[17] G. Nogay *et al.*, "25.1%-Efficient Monolithic Perovskite/Silicon Tandem Solar Cell Based on a p -type Monocrystalline Textured Silicon Wafer and High-Temperature Passivating Contacts," *ACS Energy Lett.*, vol. 4, no. 4, pp. 844–845, Apr. 2019, doi: 10.1021/acsenergylett.9b00377.
